# The Anti‐Constipation Effect of Garlic Polysaccharides: Roles of Gut Barrier Integrity, VIP Pathway, and the Microbiota‐SCFAs Axis

**DOI:** 10.1002/fsn3.71659

**Published:** 2026-03-17

**Authors:** Jingfang Li, Jiaxin Miao, Tianyi Li, Chanyuan Xie, Wentao Xu, Shimin Chang, Ran Chai

**Affiliations:** ^1^ College of Life Sciences and Food Engineering, Hebei University of Engineering Handan China; ^2^ Department of Nutrition and Health China Agricultural University Beijing China

**Keywords:** constipation, garlic polysaccharide, gut microbiota, intestinal barrier, prebiotic function

## Abstract

The global prevalence of functional constipation has witnessed a marked increase due to the imbalanced dietary pattern. The prebiotic function in vitro and the anti‐constipation efficacy of garlic polysaccharides (GP) in loperamide‐induced constipated mice were evaluated. During in vitro simulated digestion and fecal fermentation, GP showed digestive resistance properties and enhanced abundance of the probiotic bacteria *Bifidobacterium* and *Lactobacillus*. In vivo, GP significantly attenuated intestinal oxidative stress. Moreover, GP treatment dose‐dependently ameliorated constipation parameters. Notably, GP restored colonic barrier integrity: GP potently upregulated the mRNA expression of tight junction proteins ZO‐1 and occludin in the mechanical barrier and exhibited the most substantial restoration of both MUC2 and MUC4 expression levels in the mucins barrier, as well as reduced pro‐inflammatory cytokines of TNF‐α, IL‐1β, IL‐17 and elevated anti‐inflammatory IL‐10 in the immune barrier. Furthermore, GP regulated VIP‐cAMP‐PKA‐AQP3 signaling pathway, which subsequently promoted intestinal water transport and mucus secretion. GP significantly regulated the gut microbiota‐SCFAs axis by lowering the *Firmicutes/Bacteroidetes* ratio and suppressing the level of pathobiont *Desulfovibrio*. GP intervention significantly elevated total SCFAs compared to the MOD group (*p* < 0.05). These results elucidated GP's multi‐target mechanism against functional constipation via antioxidant, barrier‐repairing, VIP pathway‐regulating, and microbiota‐directed SCFAs axis.

## Graphical abstract

Garlic polysaccharides (GP) demonstrated potential against functional constipation. In vitro, GP exhibited digestive resistance properties and boosted beneficial *Bifidobacterium* and *Lactobacillus*. In constipated mice, GP alleviated constipation by reducing oxidative stress and repairing the mechanical, chemical, and immune barriers. Moreover, GP regulated the VIP‐cAMP‐PKA‐AQP3 pathway to improve water transport and mucus secretion and alleviated constipation by modulating the microbiota‐SCFAs axis.

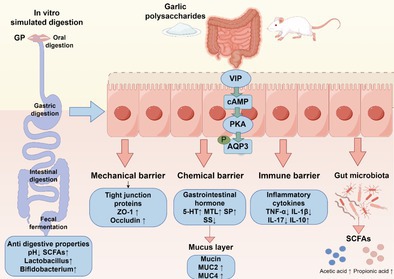

## 1 Introduction

Improvements of living standards have led to changes in diet and lifestyle, making functional constipation increasingly prevalent in both children and adults (Shi et al. 2024; Wald 2016). According to the Rome III diagnostic criteria, functional constipation refers to constipation without organic causes, with a global prevalence rate of approximately 10.1%, characterized by decreased bowel movement frequency, difficulty in defecation, reduced fecal water content, and abdominal bloating and pain (Barberio et al. 2021). Drugs or surgery are typical options for clinical treatment of constipation. However, these treatments may lead to serious complications, such as drug dependence, side effects, and withdrawal symptoms, and also place a higher financial burden on patients, more than half of whom are not fully satisfied with the efficacy of these therapies (Huo et al. 2022; Vriesman et al. 2020). Therefore, the use of natural products to develop therapeutic means has become a new research hotspot. Studies have shown that prebiotics and biostime can effectively relieve functional constipation by restoring intestinal health and enhancing intestinal movement (Wang et al. 2024).

The intestine is a selective semi permeable barrier, which mainly includes mechanical barrier, chemical barrier, immune barrier and gut microbiota. If intestinal barrier function is compromised, harmful substances entering the intestine may trigger an immune response (Gou et al. 2022). The intestinal mechanical barrier is structurally based on the intestinal mucosal epithelial cells and their intercellular junctions. It serves as a crucial barrier protecting the organism from the external environment and is often the primary target for pathogens (Kurashima and Kiyono 2017). Tight junction proteins are essential for maintaining the structural integrity of the intestinal mucosal barrier (Wells et al. 2022). Tight junction proteins such as occludin and claudin function as structural proteins, and zonula occludens‐1 (ZO‐1) acts as a functional protein. The constipation induces damage to the intestinal mechanical barrier, reducing the levels of intestinal tight junction proteins. Xie et al. (2019) found that in constipated mice, intestinal length decreased, the compactness of intestinal epithelial cell arrangement was reduced, and the expression levels of intestinal mucin, occludin, and tight junction proteins were significantly downregulated (Xie et al. 2019). Goblet cell‐derived mucins and gastrointestinal hormones are vital components of the intestinal chemical barrier. These mucins like mucin 2 (MUC2), mucin 4 (MUC4) help lubricate the gut and regulate the microbiota (Shon et al. 2022). Barrier disruption will reduce goblet cells and suppress mucins expression. Gastrointestinal hormones and neurotransmitters are useful in maintaining intestinal chemical barrier and homeostasis. By stimulating afferent neuron receptors, 5‐hydroxytryptamine (5‐HT) activates specific neuronal subtypes, altering levels of biomarkers like motilin (MTL) and substance P (SP), vasoactive intestinal peptide (VIP) and somatostatin (SS), then these alterations ultimately promoting or inhibiting intestinal motility (Bai et al. 2022; Lu et al. 2024). VIP, as an intestinal neurotransmitter, regulates intestinal water transport by activating an important signaling pathway: VIP promotes the conversion of ATP to cyclic AMP (cAMP), subsequently activating protein kinase A (PKA) and inducing phosphorylation of aquaporin‐3 (AQP3). Dysfunction of the VIP‐cAMP‐PKA‐AQP3 pathway contributes to functional constipation (Dimidi et al. 2017; Ren et al. 2017). The intestine is the largest immune organ of the body, harboring approximately 70% of the body's immune defenses (Zouali 2021). The intestinal immune barrier, involving immunoglobulins and immune factors, protects against functional constipation. Anti‐inflammatory cytokines including interleukin‐4 (IL‐4), interleukin‐10 (IL‐10), interleukin‐11 (IL‐11), and interleukin‐13 (IL‐13) maintain intestinal homeostasis. Upon disruption, pro‐inflammatory cytokines including tumor necrosis factor alpha (TNF‐α), interleukin‐1 beta (IL‐1β), interleukin‐6 (IL‐6), and interleukin‐17 (IL‐17) are upregulated, compromising physiological equilibrium (Hossen et al. 2022). Wang et al. (2022) demonstrated *Bifidobacterium* alleviated inflammation and prevents barrier damage in constipated mice by downregulating IL‐1β and TNF‐α (Wang et al. 2022). The immune barrier function plays a protective role in constipated individuals, and its disruption is implicated in constipation pathogenesis. Research on natural bioactive components repairing the intestinal barrier to alleviate constipation is well‐documented. Filippone et al. (2022) demonstrated that xyloglucan alleviated constipation‐predominant irritable bowel syndrome by restoring occludin and ZO‐1 expression, as well as repairing intestinal integrity (Filippone et al. 2022). Shatianyu (*Citrus grandis L*. Osbeck) alleviated loperamide‐induced constipation in mice by enhancing colonic 5‐HT secretion and downregulating TNF‐α and IL‐1β, consequently repairing the intestinal chemical barrier (Deng et al. 2024). Furthermore, inflammatory cytokines also lead to aberrant expression of tight junction proteins, including ZO‐1 and claudin‐1, highlighting the close interrelation among intestinal barriers. However, the underlying mechanisms of the synergistic barrier require further investigation to fully elucidate the intestinal barrier‐constipation relationship.

Polysaccharides, as a class of important pharmacologically active prebiotics, are safe, low‐toxicity substances with various biological activities including antioxidant, anti‐inflammatory, anti‐tumor, lipid‐lowering, and gut health (Premarathna et al. 2024; Xu et al. 2025). The intake of polysaccharides showed the ability to improve intestinal barriers and change the diversity of gut microbiota, and the degradation and utilization of indigestible polysaccharides by gut microbiota can produce favorable metabolites to reduce the risk of intestinal diseases (Keung et al. 2025). Polysaccharides were fermented by gut microbiota into short‐chain fatty acids (SCFAs), which could reduce the inflammation of the gastrointestinal tract, modulate the intestinal pH, inhibit the proliferation of harmful bacteria, and improve the immune function of the intestinal mucosa (Gu et al. 2020). Liu et al. (2022) discovered that *Cistanche deserticola* polysaccharides alleviate functional constipation in rats by modulating the gut microbiota and ameliorating 16 potential FC‐related metabolites (Liu et al. 2022).

Garlic ( *Allium sativum* L.) belongs to the alliaceae family and is widely used worldwide as a condiment because of its unique fragrance (Pacholczyk‐Sienicka et al. 2024). It is also valued for its various pharmaceutical effects, including bacteriostasis, antiphlogosis, anti‐cardiovascular diseases, and anti‐tumor properties (Zhang, Li, et al. 2024). Garlic polysaccharides (GP) are the essential active constituents of garlic, accounting for more than 75% of garlic bulb dry matter (Ritota et al. 2012). Initially classified as inulin due to its ⊎‐2,1 linked fructan structure, GPs were neglected in research and development. Further analysis revealed that GPs have (2 → 6)‐linked ⊎‐D‐Fruf branched chains with relatively low molecular weight, providing high water solubility and suitability for food processing, attracting more research attention. Wu et al. (2024) revealed that GP reversed histopathological damage to the small intestine and spleen and increased fecal SCFAs. Zhan et al. (2022) further revealed that GP attenuated constipation and colitis by enhancing intestinal barrier function. The therapeutic potential of GP has garnered attention; however, the specific mechanisms underlying their alleviation of functional constipation remain insufficiently explored. Particularly, the underlying mechanisms, and how the different pathways work in concert to alleviate constipation, require further in‐depth investigation.

Therefore, the objectives of the present study were to (i) evaluate the interventional effects of garlic polysaccharides by employing loperamide hydrochloride to induce a functional constipation model in BALB/c mice; (ii) assess the impact of garlic polysaccharides on the intestinal barrier, VIP pathway, gut microbiota, and SCFA metabolism for functional constipation improvement, and investigate the underlying mechanisms. This research will provide essential data for utilizing natural plant polysaccharides as a future improvement of functional constipation.

## 2 Materials and Methods

### 2.1 Materials and Chemicals

GP (purity > 97%) and lactulose are provided by Chenguang Biotech Group CO. LTD. The preparation method of GP was detailed in patent CN 112812196B. Specifically, GP was extracted from garlic using PBS as the solvent at a solid‐to‐liquid ratio of 1:3 (*w*/*w*). After extraction, the mixture was centrifuged at 4200 rpm to collect the supernatant, and the flocculant and activated carbon were added to the supernatant. The mixture was stirred, filtered, and then treated with a flocculant consisting of potassium hydroxide and calcium hydroxide. Flocculation was performed at 80°C for 2 h. The garlic extract was subjected to decolorization and desalination using anion‐exchange chromatography and cation exchange chromatography columns. The decolorized and desalted garlic extract was concentrated by a nanofiltration membrane. The resulting purified extract was spray‐dried to obtain GP powder. The structural characterization of GP was previously reported (shown in Figure S1) (Xie et al. 2023).

The chemical reagents were analytical grade purchased from Xilong Scientific (Guangdong, China).

### 2.2 In Vitro Simulated Digestion of GP

An in vitro three‐stage digestion model simulating saliva, gastric, and small intestinal fluids was established based on the method of Brodkorb et al. (2019) with modifications. A GP sample solution (15 mg/mL) was mixed with simulated saliva at a 1:1 (*v*:*v*) ratio and incubated at 37°C to simulate oral digestion. After 5 min, the pH of the salivary digest was adjusted to 3.0. This mixture was then combined with pre‐warmed (37°C) simulated gastric fluid at a 1:1 (*v*:*v*) ratio and incubated at 37°C to simulate gastric digestion. Following 6 h of gastric digestion, the pH of the saliva‐gastric digest was adjusted to 7.0 and mixed with simulated small intestinal fluid at a 10:3 (*v*/*v*) ratio. This mixture was incubated at 37°C for 6 h to simulate intestinal digestion. Samples were collected after 0 and 5 min of simulated salivary digestion and after 1, 2, 4, and 6 h of both simulated gastric and small intestinal digestion phases. Collected samples were immediately subjected to enzyme deactivation by heating at 100°C for 10 min, followed by centrifugation at 4000 rpm for 10 min. The resulting supernatant was collected for subsequent analysis.

### 2.3 Microbial Fermentation of GP by Human Feces

Human fecal inoculum was prepared from 6 healthy volunteers (3 males, 3 females; aged 22–25 years; no intestinal disease history for 6 months, no probiotics/antibiotics for at least 4 weeks). Feces were diluted 1:9 (*w*/*v*) with sterile PBS (pH 7.0), vortexed, centrifuged at 1000 rpm for 10 min, and the supernatant was collected. The basal medium was prepared according to the method described by Liu et al. (2021) with some modifications. One liter of basal medium contained the following components: 2.5 g peptone, 4 g yeast extract, 0.1 g sodium chloride, 0.04 g K_2_HPO_4_, 0.04 g KH_2_PO_4_, 0.01 g MgSO_4_, 0.01 g CaCl_2_, 2 g NaHCO_3_, 2 g mucin, 0.5 g bile salts, 0.46 g L‐cysteine hydrochloride, 2 mL Tween‐80, 50 mL hemin solution, 0.015 g vitamin K_3_, and 4.95 mL resazurin solution. The mixture was adjusted to pH 7.0 using 0.5 M HCl, sterilized by autoclaving at 121°C for 15 min, and cooled prior to use. The in vitro fermentation study included four groups: (1) blank group, (2) glucose group (positive control), (3) GP group, and (4) inulin group (positive control). Each tube in the blank group received 10 mL of fecal supernatant and 20 mL of basal medium. Each tube in the GP group received 10 mL of fecal supernatant and 20 mL of basal medium supplemented with 1% (*w*/*v*) GP. Each tube in the positive control group received 10 mL of fecal supernatant and 20 mL of basal medium supplemented with 1% (*w*/*v*) inulin or glucose. Tubes were immediately transferred to an anaerobic chamber. All fermentation tubes were incubated anaerobically at 37°C. After 12, 24, and 48 h of incubation, fermentation mixtures were centrifuged at 12,000 rpm for 10 min. The resulting supernatant and pellet were separated. The pellet was stored at −80°C for subsequent microbial analysis, and the supernatant for subsequent metabolite analysis. The samples were applied for the detection of pH, SCFAs, OD_600_, and growth of *Bifidobacterium* and *Lactobacillus* during fermentation.

### 2.4 Animal Experiments

A total of 60 male BALB/c mice (aged 7 weeks) were obtained from SiPeiFu Biotechnology Co. Ltd. (Beijing, China) and the Certificate of Quality is No. SCXK (Beijing) 2019‐0010. All animal procedures and testing were performed in compliance with the national legislation on the use and care of laboratory animals and approved by the experimental animal ethics committee of the affiliated hospital of Hebei University of Engineering (Permit Number: IACUC‐Hebeu‐2023‐00287). All mice were kept in an SPF‐level laboratory animal room and housed in a room maintained at constant temperature of 23°C ± 1°C and 50% ± 5% relative humidity condition with a 12‐h light/dark cycle. After 1‐week acclimation period, the mice were randomly divided into 6 groups (*n* = 10, as shown in Figure 2A: [1] control group [CON], [2] constipation model group [MOD], [3] GP low‐dose group [GPL, with a gavage dose of 1.25 g/kg BW GP], [4] GP medium‐dose group [GPM, with a gavage dose of 2.5 g/kg BW GP], [5] GP high‐dose group [GPH, with a gavage dose of 5 g/kg BW GP], [6] positive control group [PC, with a gavage dose of 2.5 g/kg BW lactulose]). Except for mice in the CON group receiving saline as control, all other group mice received loperamide at 10 mg/kg BW via oral gavage to induce constipation daily at 9:00 a.m. After 1 h, mice were treated with GP (1.25, 2.5, 5 g/kg BW) or lactulose (2.5 g/kg BW) daily for 3 weeks (Xie et al. 2024). All mice were treated orally by gavage once a day. The food and water were available ad libitum. Body weights and food intake were recorded once every 3 days. After 3 weeks of intervention, mice were fasted overnight before being euthanized. Blood samples were collected and then kept at room temperature for 2 h to ensure complete clotting before centrifugation at 4°C, 5000 rpm (Thermo Scientific, MA, USA) for 10 min to obtain the serum sample. Mice were then necropsied and the colon tissue was extracted for analysis. Colon tissue was immersed in a 4% paraformaldehyde solution (*w*/*v*). The colon tissue and colon contents were rapidly frozen in liquid nitrogen and stored at −80°C for analysis.

### 2.5 The Antioxidant Assay In Vitro and In Vivo

GP solutions were formulated at mass concentrations ranging from 0.01 to 1.0 mg/mL, with vitamin C (VC) solutions at identical concentration ranges serving as positive controls. Experimental procedures of ABTS^+^ radical scavenging activity, DPPH• radical scavenging activity, hydroxyl radical test, and superoxide anion radical scavenging assay were measured according to the method described by Li et al. (2019) with minor modifications. The contents of superoxide dismutase (SOD), glutathione peroxidase (GSH‐Px), catalase (CAT), and malondialdehyde (MDA) in colon tissue homogenate were also determined according to the instructions of the corresponding assay kits (Nanjing Jiancheng Bioengineering Institute, Nanjing, China).

### 2.6 Time of the First Blank Fecal Defecation

After a 12 h fast following the end of the medication treatment, mice were intragastrically administered 0.2 mL of activated carbon solution and GP. Following gavage, mice were individually housed in clean cages lined with absorbent paper under fasting conditions (water ad libitum). The time was recorded after observing the discharge of the first black stool.

The activated carbon solution was prepared by dissolving 50 g of arabic gum in 400 mL of distilled water under continuous stirring and boiling until a transparent solution was obtained. Subsequently, 25 g of activated charcoal powder was thoroughly mixed into the solution. After cooling to room temperature, the mixture was diluted with an additional 500 mL of distilled water and stored at 4°C (Liu et al. 2022).

### 2.7 Stool Pellet Number and Moisture Content

On the 20th day of the animal test, each mouse was put into a clean cage separately, the fresh feces discharged within 5 h were collected, and the total fecal particles discharged were recorded. The wet weight of fecal samples was measured and recorded. Subsequently, the samples were dried in an oven maintained at 90°C until constant weight was achieved, after which the dry weight was determined and calculated.

### 2.8 Intestinal Transit Rate

At the end of the animal experiment, all mice underwent a 16‐h overnight fast with free access to water, and the mice were given 0.2 mL of activated charcoal meal orally. After 15 min, mice were euthanized via cervical dislocation under anesthesia. The abdominal cavity was immediately opened to expose the mesentery, and the entire small intestine was excised and placed on a tray. The intestine was gently stretched into a straight line, and the transmission distance of the activated carbon powder and total intestinal length were measured to calculate the intestinal transit rate.

$$\text{The intestinal transit rate}=\frac{\text{the transmission distance of the activated carbon powder}}{\text{total intestinal length}}\times 100\%$$

### 2.9 Morphology of Colon Tissues

Colon tissues were fixed with 4% paraformaldehyde and embedded in paraffin. The 5‐μm sections were prepared and stained with hematoxylin and eosin (H&E). The physiology of hepatic tissues was visualized using an inverted microscope (Olympus, Tokyo, Japan) under a magnification of 200×.

### 2.10 Biochemical Analysis

Gastrointestinal active peptides and neurotransmitters in serum including 5‐HT, SP, MTL, SS, and VIP, as well as inflammatory factors including TNF‐α, IL‐1β, IL‐10, and IL‐17 were quantified according to the instruction of commercial kits (Nanjing Jiancheng Bioengineering Institute, Nanjing, China).

### 2.11 RT‐qPCR Analysis

The RT‐qPCR was determined with TB Green Premix Ex Taq (Tli RNaseH Plus, Takara BIO, Shiga, Japan). Total RNA was extracted using Trizol reagent according to the manufacturer's instructions and quantified by a NanoDrop ND‐1000 spectrophotometer. RNA was reverse transcribed into cDNA using a Prime Script RT reagent kit (Takara, Japan). The mRNA level was quantified by the 2^−ΔΔCt^ method. The forward and reverse sequences of qPCR primers are shown in Table S1. The PCR reaction cycle was set as 95°C for 30 s, 35 cycles at 95°C for 5 s, and 60°C for 30 s. The GAPDH was used as the housekeeping gene and relative expression level was shown as fold changes relative to the CON group.

### 2.12 Gut Microbe 16S rRNA Sequencing

Fecal gut microbiota profiles were determined by 16S rRNA amplicon sequencing. The microbial genome DNA was extracted with the magnetic soil and stool DNA kit according to manufacturer instructions (Tiangen Biotech Co. Ltd., Beijing, China). DNA concentrations were normalized to 1 ng/μL with sterile water, and the diluted DNA was amplified using the specific primer pair 515F‐907R targeting the V4‐V5 hypervariable regions of the 16S rRNA gene. Equal volumes of 1× loading buffer and PCR products were mixed and electrophoresed on 2% agarose gels. Samples exhibiting bright primary bands within the 400–450 bp range were selected for purification using the GeneJET Gel Extraction Kit. Sequencing libraries were constructed with the NEB Next Ultra DNA Library Prep Kit for Illumina (NEB, USA). Library quality was assessed using a Qubit 2.0 Fluorometer and Agilent Bioanalyzer 2100 system. Purified libraries were sequenced on the Illumina MiSeq platform (Illumina, USA), and raw data were demultiplexed and analyzed through a cloud‐based bioinformatics platform. Predicted metagenomic functions were analyzed by correlating the 16S rRNA gene sequences with the Clusters of Orthologous Groups (COG) database using PICRUSt2.

### 2.13 The Contents of SCFAs in Feces

The contents of SCFAs in feces were determined according to the method of Lou et al. (2024) and Yao et al. (2022) with modifications (Lou et al. 2024; Yao et al. 2022). Frozen colon fecal samples (50 mg) were homogenized in 1 mL of PBS. The mixture was centrifuged at 10,000 rpm for 30 min at 4°C. Under ice‐bath conditions, the supernatant was sequentially treated with 1.2 mL of anhydrous ethanol, 0.1 mL of concentrated sulfuric acid, and 1 mL of *n*‐hexane. The mixture was transferred to a constant‐temperature shaker and incubated at 60°C for 1 h with continuous agitation at 200 rpm. Following incubation, anhydrous CaCl_2_ was added as a dehydrating agent. The sample was centrifuged at 12,000 rpm for 10 min, and the clarified supernatant was filtered through a 0.22‐μm nylon membrane.

A GC–MS system (7890B‐7000D, Agilent Technologies, Santa Clara, USA) equipped with a HP‐FFAP capillary column (30 m × 0.25 mm × 0.25 μm) was used to determine the contents of SCFAs in feces. The chromatographic separation employed the following temperature program: initial oven temperature 80°C (hold 1 min), ramped to 120°C at 10°C min^−1^, then to 150°C at 5°C min^−1^, followed by a final increase to 250°C at 25°C min^−1^ (maintained for 2 min), achieving complete elution within 15 min. Mass spectra were acquired in electron impact ionization (EI) mode at 70 eV with selected ion monitoring (SIM). The injector and MS transfer line temperatures were set to 250°C and 280°C, respectively. Quantification of SCFA concentrations was achieved by normalizing the peak area of each SCFA to that of the internal standard.

### 2.14 Statistical Analysis

All data were presented as mean ± SEM (standard error of the mean). Animal experiments were performed with 10 biological replicates and each sample was tested 3 times at least for technical replicates. Statistical analysis was performed using SPSS software (Version 25.0, SPSS Inc., Chicago, IL, USA). Graph bars marked with different letters on top represent statistically significant results (*p* < 0.05) based on One‐way ANOVA analysis followed by *Duncan*'s test.

## 3 Results and Discussion

### 3.1 Dynamic Variations of GP During Simulated Digestion and Fecal Fermentation In Vitro

The total sugar content of GP and changes in reducing sugar contents after in vitro simulated oral, gastric, and intestinal digestion were shown in Table S2. The total sugar content of GP was 14.2713 ± 0.0182 mg/mL, while the initial reducing sugar content was 0.4493 ± 0.0121 mg/mL. After 5 min of simulated salivary digestion, the reducing sugar content was 0.4553 ± 0.0147 mg/mL, showing no significant difference compared to the undigested sample (*p* > 0.05). Following 6 h of simulated gastric digestion, the reducing sugar content significantly increased to 0.5199 ± 0.0133 mg/mL (*p* < 0.05). This increase is likely attributed to the acidic environment of the gastric fluid promoting the cleavage of glycosidic bonds, thereby releasing more reducing sugars (Liu et al. 2021). After a subsequent 6 h of simulated intestinal digestion, the reducing sugar content reached 0.5364 ± 0.0126 mg/mL, which was not significantly different from the level observed after gastric digestion (*p* > 0.05). There was no significant change of GP in the simulated digestion, showing its digestive resistance properties, indicating that GP may enter the colon and be utilized and fermented by gut microbiota, demonstrating its potential to exert prebiotic effects.

**FIGURE 1:**
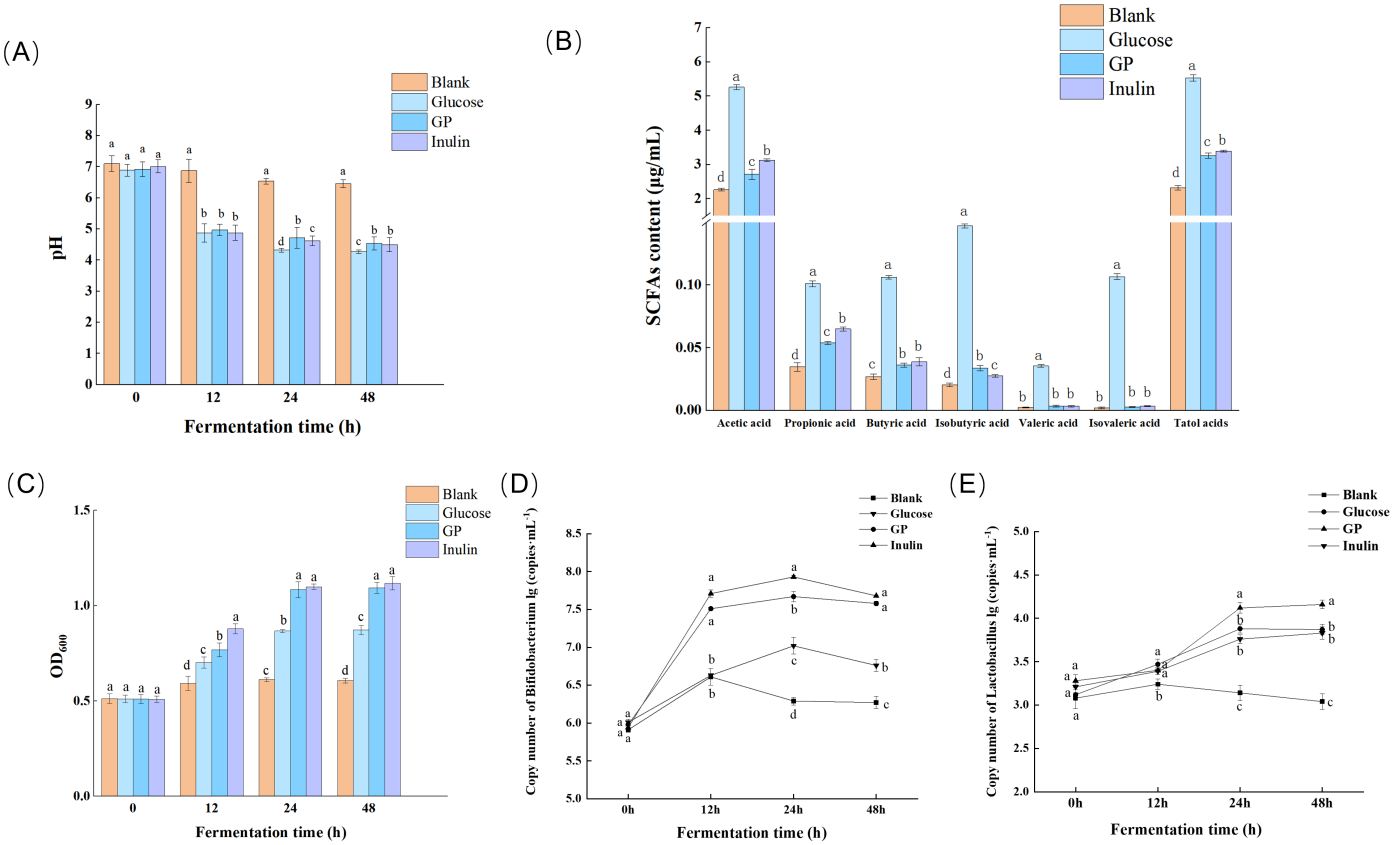
Changes in simulated digestion and fecal fermentation of GP in vitro. (A) pH of the fermentation solution at different times; (B) SCFAs contents in the fermntation solution; (C) OD_600_ value of fermentation solution; (D) Growth of *Bifidobacterium* during fermentation; (E) Growth of *Lactobacillus* during fermentation. Data represent the mean ± SEM (*n* = 6). Bars with various lowercase letters under the same time conditions differ significantly using one‐way ANOVA followed by the Duncan's test, *p* < 0.05.

Figure 1A illustrates changes in the pH of the fermentation broth during fecal fermentation, indicating the utilization of GP by gut microbiota. At the initial stage (0 h), the pH values across all groups showed no significant differences (*p* > 0.05). After 12 h of fermentation, the pH values in the glucose, GP, and inulin groups were significantly lower than those in the blank group (*p* < 0.05). This suggested rapid microbial utilization of glucose, GP, and inulin during this period, generating substantial acidic metabolites such as SCFAs, which consequently caused a sharp pH decline. Between 12 and 48 h, the rate of pH decreased slowly in all groups. Following 48 h of simulated fecal fermentation, the final pH of all test groups was lower than that of the blank group. The pH of the blank group was 6.46 ± 0.13, while the pH of the glucose, GP, and inulin groups decreased to 4.27 ± 0.02, 4.53 ± 0.01, and 4.50 ± 0.01, respectively. Notably, no significant difference was observed between the GP and inulin groups (*p* > 0.05).

Figure 1B shows the concentrations of SCFAs after fecal fermentation in vitro. The acetic acid was the most abundant SCFAs, with significantly higher levels than the other SCFAs measured. Due to the lack of an added carbon source, the total SCFAs content in the blank group was significantly lower than that in the glucose, GP, and inulin groups (*p* < 0.05). Glucose, serving as a readily available carbon source, produced significantly higher total SCFAs concentrations than the other groups (*p* < 0.05), attributable to its direct and rapid microbial utilization. The GP group exhibited significantly higher total SCFAs levels than the blank group. The isobutyric acid concentration in the GP group was significantly higher than in the inulin group (*p* < 0.05), and the concentrations of butyric acid, valeric acid, and isovaleric acid in the GP group showed no significant difference compared to the inulin group (*p* > 0.05). Liu et al. (2025) confirmed that polysaccharides showed the ability to regulate the metabolism of SCFAs.

Figure 1C depicts the OD_600_ values, reflecting the proliferation of gut microbiota during fecal fermentation. At 0 h, no significant differences in OD_600_ were observed among the 4 groups (*p* > 0.05). After 12 h of in vitro fecal fermentation, the OD_600_ values in the glucose, GP, and inulin groups were significantly higher than in the blank group. By 48 h, the OD_600_ values in both the GP and inulin groups were significantly higher than those in the other groups (*p* < 0.05), with no significant difference observed between the GP and inulin groups (*p* > 0.05). During fecal fermentation, the growth dynamics of *Bifidobacterium* and *Lactobacillus* are shown in Figure 1D,E. *Bifidobacterium* exhibited rapid growth from 0 to 12 h, while beyond 12 h, the growth rate slowed in the inulin, GP, and Glucose groups, followed by a declining trend after 24 h. At 48 h, the *Bifidobacterium* levels in both the inulin and GP groups were significantly higher than in the glucose group (*p* < 0.05), indicating the prebiotic effect of GP and inulin in promoting *Bifidobacterium* proliferation. Overall growth of *Lactobacillus* was slow during the initial 0–12 h period. Between 12 and 24 h, accelerated growth occurred in the GP, glucose, and inulin groups. Growth then plateaued between 24 and 48 h. At the end of the 48 h fermentation, the *Lactobacillus* level in the inulin group was significantly higher than in all other groups. No significant difference was observed between the GP and glucose groups (*p* > 0.05). The observed differences in specific SCFAs profiles between GP and inulin are primarily attributed to their structural distinctions. GP possesses β‐2,6‐fructosidic side chains, leading to variations in fermentation kinetics and metabolite production with inulin. The results indicated that GP demonstrated significant potential as a prebiotic, enhancing probiotic growth (particularly *Bifidobacterium*), and promoting intestinal health. These findings align with previous in vitro fermentation studies of polysaccharides (Fu et al. 2023; Wu et al. 2021; Wu et al. 2022).

### 3.2 GP Alleviated Constipation

**FIGURE 2:**
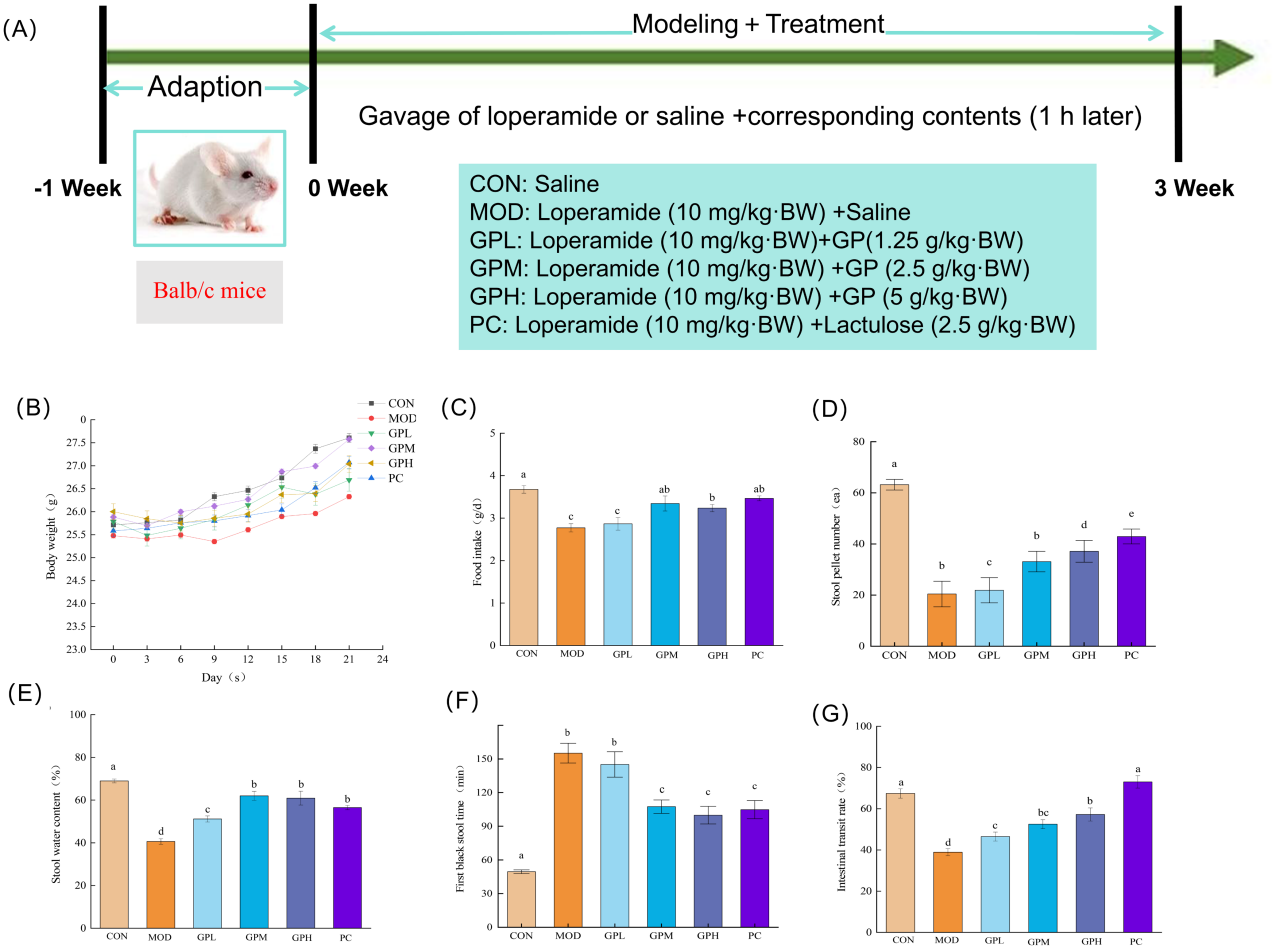
Effects of GP treatment on the physiological indexes and defecation‐related parameters in the constipated mice. (A) Scheme of animal experiment; (B) body weight; (C) food intake; (D) stool pellet number; (E) stool water content; (F) first black stool time; (G) intestinal transit rate. Data represent the mean ± SEM (*n* = 10). Bars with various lowercase letters differ significantly using one‐way ANOVA followed by the Duncan's test, *p* < 0.05.

As illustrated in Figure 2B, all loperamide hydrochloride‐modeled groups (GPL, GPM, GPH, and PC) exhibited body weight reduction during days 0–3, whereas the CON group remained unaffected. At the end of intervention, the MOD group maintained the lowest body weight, while GP and lactulose interventions effectively ameliorated its slow weight recovery trend. Compared with the CON group, the MOD group displayed significantly reduced daily food intake (Figure 2C, *p* < 0.05). While GPM, GPH, and PC groups exhibited a significant increase in daily food consumption (*p* < 0.05), particularly the GPM and PC groups which restored intake levels to those of the CON group (*p* > 0.05). The persistently lower body weight in MOD group mice was attributed to loperamide hydrochloride‐induced functional constipation, which compromised their health status and suppressed appetite‐driven feeding activity.

As illustrated in Figure 2D–G, the MOD group showed a notable reduction in stool pellet number, stool water content, and intestinal transit rate, as well as an increase in first black stool time (*p* < 0.05), indicating the constipation model was established successfully. In contrast, oral administration of GP elicited significant amelioration of constipation‐related parameters in mice. In detail, stool pellet number showed a marked elevation in GP groups with a dose‐dependent relationship (*p* < 0.05, Figure 2D). The stool water content of MOD group mice exhibited a significant 41.09% reduction compared with the CON group (*p* < 0.05, Figure 2E). The results of GPL, GPM, and GPH groups demonstrated remarkable increases in stool water content relative to the MOD group (*p* < 0.05), with the GPM group achieving the highest fecal wet weight, showing a 60.79% elevation compared to the CON group. Compared with the CON group, the first black stool time of the MOD group was significantly prolonged from 49.5 min to 157.2 min (*p* < 0.05, Figure 2F). Importantly, GPM and GPH demonstrated significant reductions in the first black stool time (*p* < 0.05). Notably, the GPH group exhibited a remarkably shortened duration of 99.9 min, which was shorter than the 104.8 min observed in the PC group. The intestinal transit rate in CON mice was 67.40% ± 2.26%, whereas MOD mice exhibited a significant 42.28% reduction (*p* < 0.05, Figure 2G). GP treatment dose‐dependently increased the intestinal transit rate compared to the MOD group (*p* < 0.05). The results were consistent with the findings of Hu et al. (2025). The results of the present study demonstrated that GP administration stimulated intestinal peristalsis in mice, thereby ultimately ameliorating fecal constipation symptoms. In addition, the restorative effects of GP on stool water content and fecal pellet number were comparable to those reported for some prebiotic polysaccharides, such as polysaccharide extracted from peony seed (He et al. 2025). This suggested that GP may possess a distinct potency in rapidly normalizing multiple core parameters of intestinal dysmotility, highlighting its potential as a complementary dietary intervention for functional constipation.

### 3.3 GP Exhibited Potent In Vitro and In Vivo Antioxidant Activity

**FIGURE 3:**
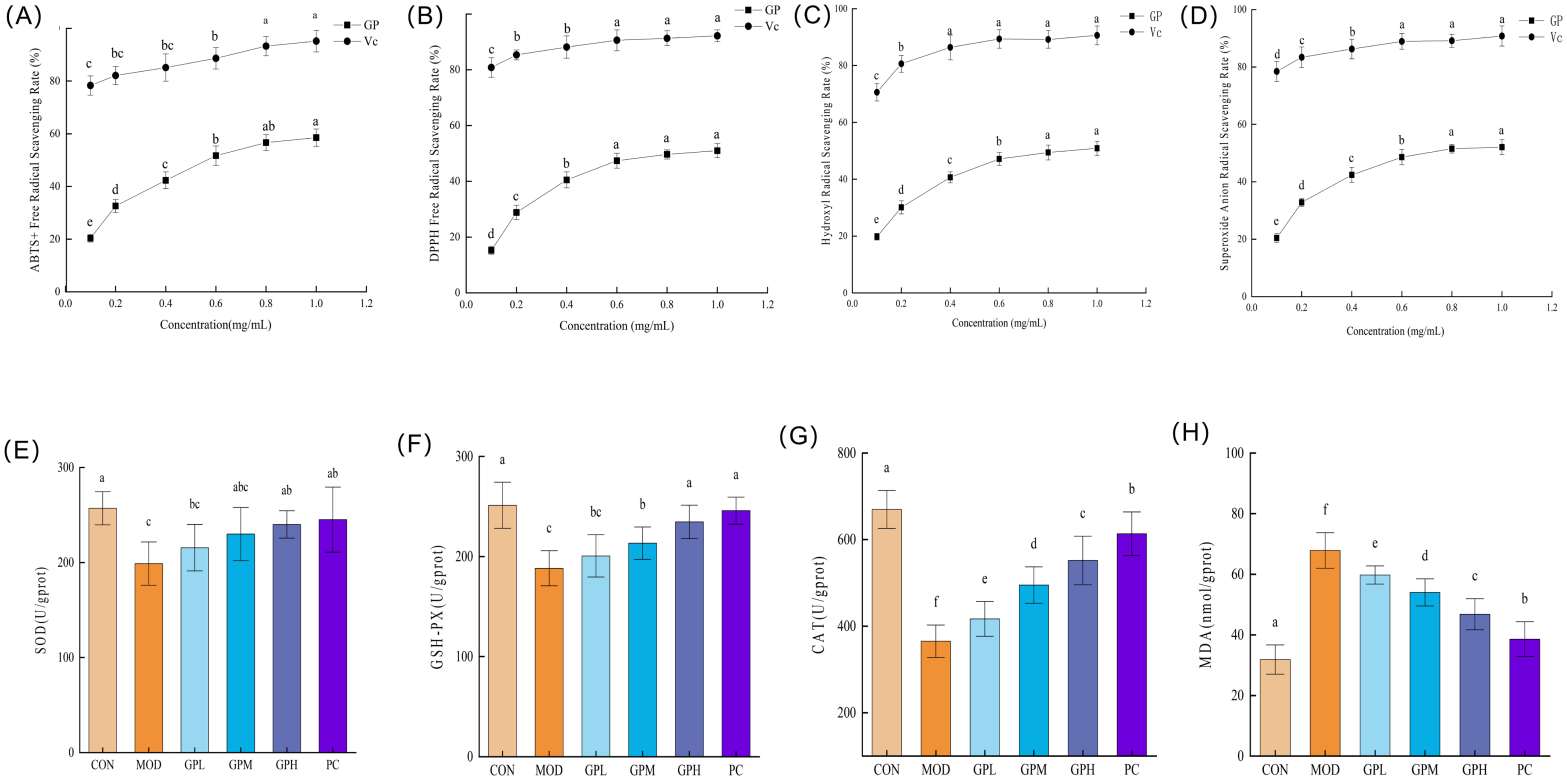
Effects of GP treatment on antioxidant indexes in vitro and in vivo. (A) ABTS^+^ radical scavenging rate; (B) DPPH radical scavenging rate; (C) hydroxyl radical scavenging rate; (D) superoxide anion radical scavenging rate; (E) SOD activity; (F) GSH‐PX activity; (G) CAT activity; (H) MDA contents. Data represent the mean ± SEM (*n* = 10). Bars with various lowercase letters differ significantly using one‐way ANOVA followed by the Duncan's test, *p* < 0.05.

Figure 3 demonstrates that the antioxidant activity of GP in vitro and in vivo. The ABTS^+^ radical scavenging activity of GP increased with concentration, reaching 55.19% at 1.0 mg/mL (Figure 3A). Similarly, the reactive functional groups in polysaccharides can reduce DPPH• radicals by donating electrons or hydrogen atoms: Within the concentration range of 0.01–1.0 mg/mL, the DPPH radical scavenging activity of GP increased with its concentration, reaching 51.48% at 1.0 mg/mL (Figure 3B). The hydroxyl and superoxide anion radical scavenging activities increased with GP concentration within the concentration range of 0.01–1.0 mg/mL, reaching 50.89% and 52.04%, respectively (Figure 3C,D) (Jie et al. 2022). Results demonstrated that GP exhibited scavenging activities against ABTS^+^, DPPH•, hydroxyl, and superoxide anion radicals. The IC_50_ values of GP were 0.73, 0.88, 0.93, and 0.79 mg/mL for ABTS^+^, DPPH•, hydroxyl, and superoxide anion radicals, respectively. The potent radical scavenging activity of GP, consistent with the findings of Shao et al. (2023), indicates potential antioxidant efficacy in vivo and lays a foundation for further investigation (Gulcin 2020).

Constipation induced significant mitochondrial oxidative stress in mouse intestines (Hao et al. 2023). As shown in Figure 3E–H, loperamide hydrochloride caused intestinal damage and oxidative stress. Consequently, the MOD group exhibited significantly reduced SOD, GSH‐Px, and CAT activities (*p* < 0.05) and elevated MDA levels (*p* < 0.05). However, GP intervention restored colonic SOD, GSH‐Px, and CAT activities in mice compared to the MOD group, particularly with the GPH group showing significant enhancement (*p* < 0.05). GP treatment also dose‐dependently reduced MDA content (*p* < 0.05), with GPH demonstrating the most pronounced reduction. In detail, relative to the CON group, the GPH intervention elevated SOD, GSH‐Px, and CAT activities by 41.10 U/g prot, 46.30 U/g prot, and 186.66 U/g prot, respectively, while reducing MDA content by 21.03 nmol/g prot. These results demonstrated GP's efficacy in enhancing intestinal antioxidant capacity and mitigating oxidative injury, consistent with findings by Song et al. (2023), where corn cob‐derived xylooligosaccharides ameliorated constipation‐induced oxidative stress through SOD/GSH‐Px elevation and MDA reduction (Song et al. 2023). Under physiological conditions, the body maintains a dynamic equilibrium between free radical generation and clearance. Following external insults, intestinal antioxidant capacity diminishes, disrupting systemic redox homeostasis. Oxidative stress, characterized by disrupted redox equilibrium and excessive reactive oxidant production, is a key pathophysiological factor (Kruk et al. 2019). This state features elevated MDA and diminished activities of GSH, CAT, and superoxide dismutase SOD. Plant polysaccharides have been proven to have antioxidant capacity (Luo, Tang, and Huang 2025). As demonstrated by Jiang et al. (2024), cistanche polysaccharides attenuated oxidative damage by reducing MDA while enhancing SOD activity and GSH levels (Jiang et al. 2024).

### 3.4 GP Restored Colonic Damage, Mechanical Barrier, and Mucus Barrier in Constipated Mice

**FIGURE 4:**
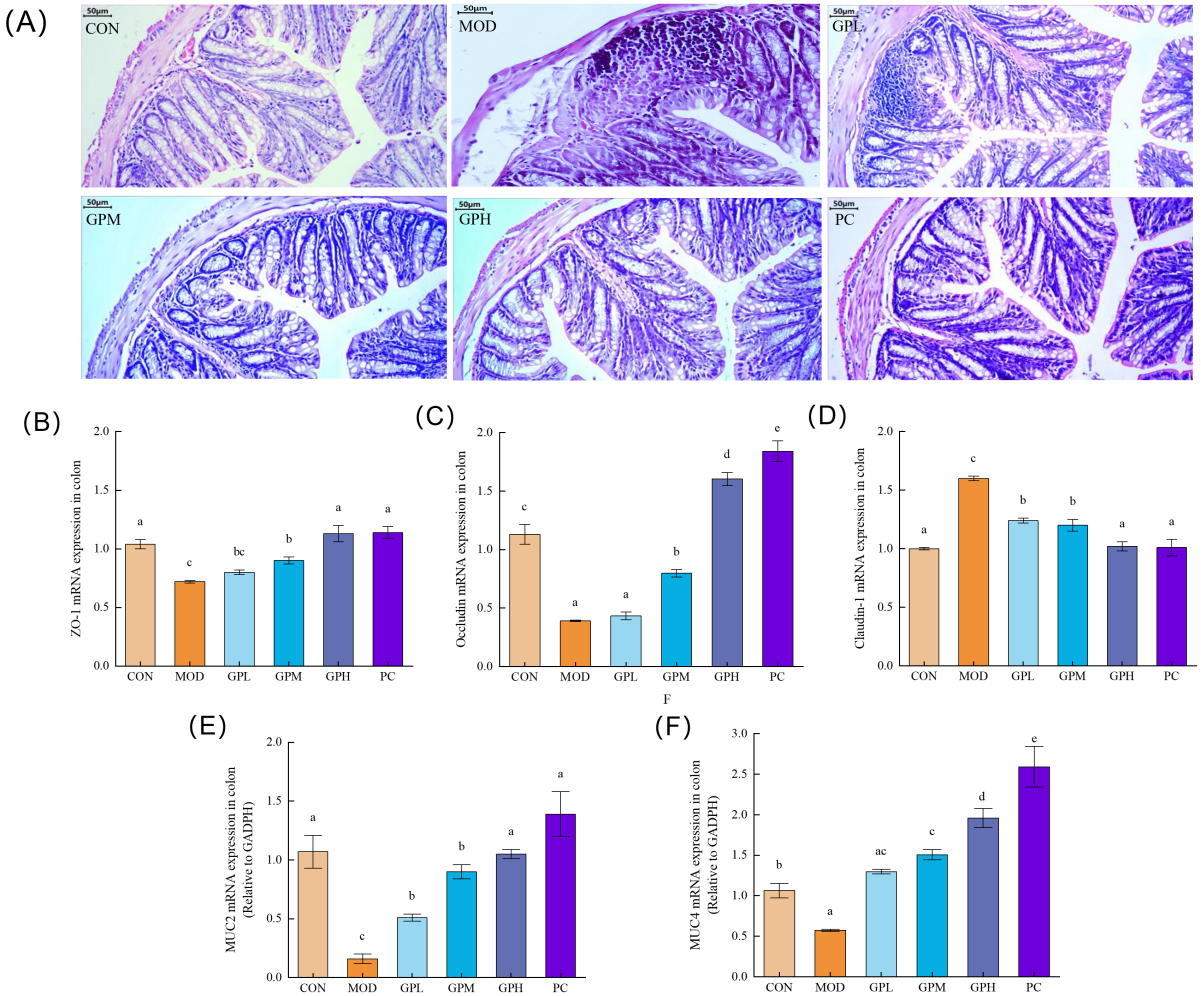
Effects of GP treatment on the H&E hepatocytes and mRNA expressions of intestinal epithelial barrier‐related adhesion molecules in the colon tissue. (A) H&E hepatocytes (200×); (B) mRNA expression of ZO‐1; (C) mRNA expression of claudin‐1; (D) mRNA expression of occludin; (E) mRNA expression of MUC2; (F) mRNA expression of MUC4. Data represent the mean ± SEM (*n* = 10). Bars with various lowercase letters differ significantly using one‐way ANOVA followed by Duncan's test, *p* < 0.05.

The histopathological alterations in colonic tissues were assessed by H&E staining, as shown in Figure 4A (200× magnification). In the CON group, mice exhibited well‐organized colonic structure with intact crypt structures and abundant goblet cells. In contrast, the MOD group demonstrated significant pathological changes including reduced goblet cell population, mucosal damage, and infiltrated neutrophil cells, indicating the disruption of the intestinal mucosal barrier. Notably, the loperamide‐induced constipated colon injuries were effectively attenuated by administration of GP characterized by partial restoration of crypt morphology, goblet cell numbers, as well as accompanied by reduced infiltrated neutrophil cells and maintained mucosal integrity. Previous studies found that constipation exhibited pronounced reduced goblet cell population, inflammatory cell infiltration and characterized by deformed shortening of the crypts, suggesting that constipation may initiate inflammatory cascades and impair intestinal barrier integrity, while the anticonstipation activity of garlic fructans were confirmed in loperamide‐induced constipated mice, with particularly significant effects on the colon (Hu et al. 2025; Li et al. 2023).

Tight junctions are critical for maintaining the integrity of the epithelial monolayer and are essential for the physical barrier function of the gut in constipation patients (Zeisel et al. 2019). The reduction of zonula occludes proteins is the most significant hallmark of mechanical barrier damage, and the subsequent passage of harmful substances triggers inflammatory responses. The primary transmembrane proteins of tight junctions include ZO‐1, occludin, and claudin‐1. As shown in Figure 4B–D, following loperamide hydrochloride intervention, the mRNA expression levels of ZO‐1 and occludin were significantly lower in the MOD group compared to the CON group (*p* < 0.05), while the mRNA expression level of claudin‐1 was significantly higher (*p* < 0.05). Notably, treatment with GP increased the mRNA expression levels of ZO‐1 and occludin, especially the GPH group exhibited the most significant effect in up‐regulating the mRNA expression levels of ZO‐1 and occludin compared to MOD group (*p* < 0.05). In detail, mRNA expression levels of ZO‐1 and occludin were upregulated to 1.13‐fold and 1.60‐fold of CON levels. This finding corroborated the study by Li et al. (2024), which demonstrated the protective effects of peach gum polysaccharide on the intestinal barrier (Li et al. 2024). Furthermore, GP exhibited superior restorative effects on ZO‐1 and occludin protein expression compared to peach gum polysaccharide.

However, the observed increase in claudin‐1 expression in the MOD group after intestinal injury may stem from distinct regulatory mechanisms among different zonula occludes proteins in response to pathological stress. Occludin primarily governs tight junction assembly and maintenance, ZO‐1 as a cytoplasmic scaffolding protein anchors transmembrane tight junction proteins to the actin cytoskeleton, providing the function of structural stability and signal transduction (Schwayer et al. 2019; Zeisel et al. 2019). Under inflammatory and oxidative stress conditions, occludin and ZO‐1 demonstrate heightened susceptibility to degradation, leading to their significant downregulation. Concurrently, the intestinal epithelium may initiate compensatory upregulation of alternative components of tight junction such as claudin‐1 to partially preserve barrier function and limited pathological intestinal hyperpermeability and subsequent tissue damage. Similar results were also observed in the study of Fernández‐García et al. (2024), where claudin‐1 expression was significantly upregulated under obesity‐related chronic inflammatory and metabolic stress. Consequently, our findings demonstrated that GP treatment effectively restored the mRNA expression levels of zonula occludes proteins to physiological levels, facilitating the recovery of intestinal physical barrier integrity. This physical barrier restoration contributed to constipation amelioration and promoted gastrointestinal homeostasis.

The mucus layer formed by mucins serves as the defense in the intestinal barrier, preventing direct contact of antigens, toxins, and bacteria with epithelial cells (Senapati et al. 2010). MUC2, a secretory mucin, is the predominant mucin component in the colon. It forms dimers through disulfide bonds, undergoes glycosylation in the Golgi apparatus, and is stored as secretory granules in goblet cells. Upon specific stimuli, MUC2 is released and cross‐linked to establish a robust gel‐like mucus network (Camilleri 2019). In constipation, MUC2 expression is significantly reduced, leading to dysfunction of the mucus barrier. MUC4, membrane‐bound mucins, which are Type I transmembrane proteins containing a transmembrane domain, function in environmental sensing, signal transduction, and pathogen adhesion to maintain intestinal microecological balance. Reduced MUC4 expression in colorectal cancer patients has been associated with tumor development and progression (Grondin et al. 2020). In the present study, the gene expression revealed significant downregulation of MUC2 and MUC4 in the MOD group compared to the CON group (*p* < 0.05, Figure 4E,F), suggesting compromised mucin biosynthesis during constipation. GP administration effectively reversed this suppression, with all dose treatment groups displaying upregulated mucin gene expression (*p* < 0.05). The high‐dose GPH group exhibited the most substantial restoration of both MUC2 and MUC4 expression levels. In detail, the GPH group upregulated the gene expression of MUC2 and MUC4 to 1.05‐fold and 1.90‐fold compared to the CON group, respectively. The results align with findings from Kim et al. (2023), where snail mucin heteropolysaccharide improved loperamide hydrochloride‐induced constipation by increasing colonic MUC2 and MUC4 levels to 1.35‐ and 1.50‐fold, respectively (Kim et al. 2023).

### 3.5 Modulatory Effects of GP on Gastrointestinal Hormone Regulation, Neurotransmitter Dynamics, and Inflammatory Cytokines in Serum

**FIGURE 5:**
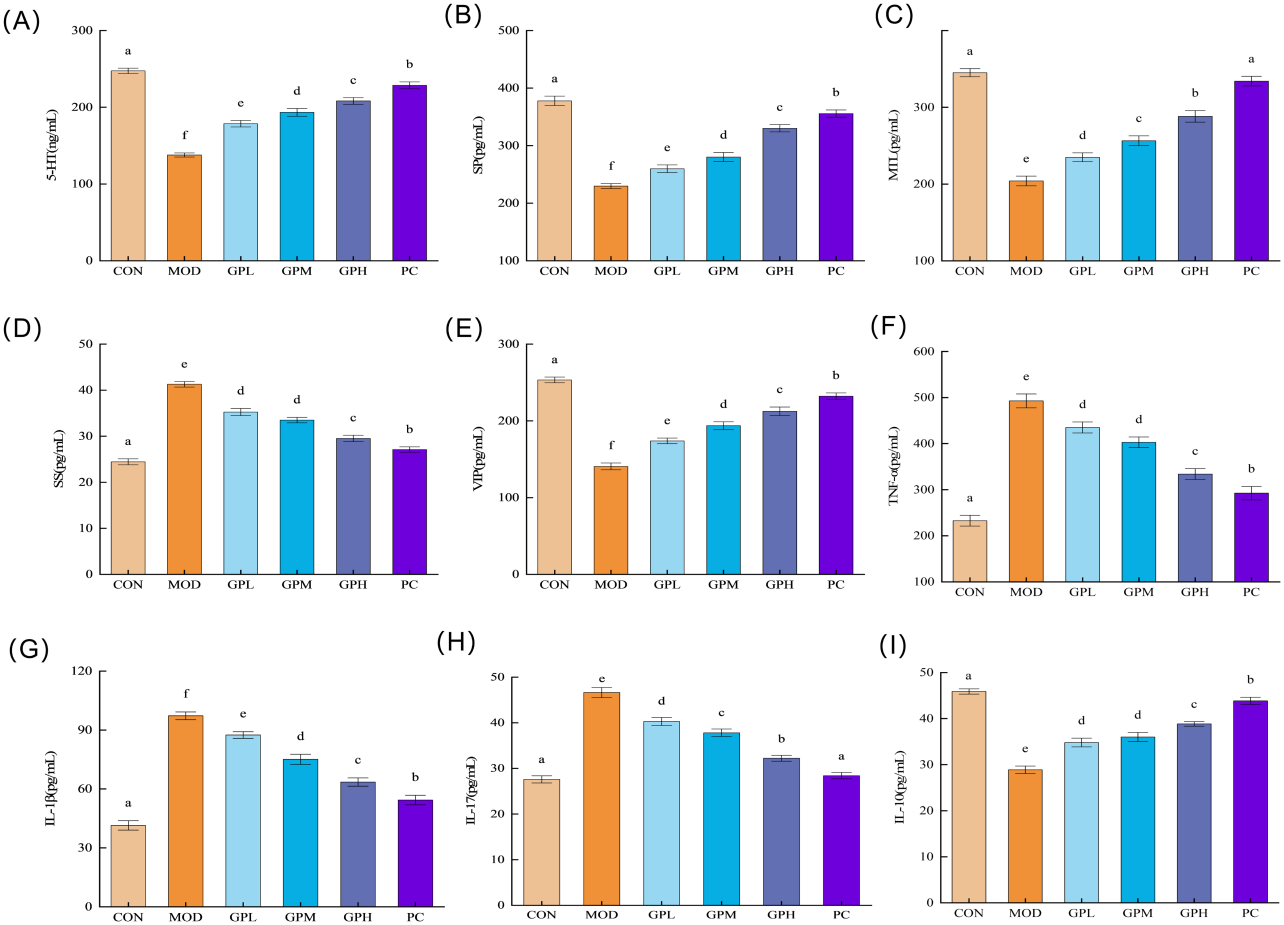
Modulatory effects of GP on gastrointestinal hormone regulation, neurotransmitter dynamics, and inflammatory cytokines in serum of the constipated mice. (A) 5‐HT; (B) SP; (C) MTL; (D) SS; (E) VIP; (F) TNF‐α; (G) IL‐1β; (H) IL‐17; (I) IL‐10. Data represent the mean ± SEM (*n* = 10). Bars with various lowercase letters differ significantly using one‐way ANOVA followed by Duncan's test, *p* < 0.05.

Gastrointestinal motility is regulated by the central nervous system (CNS) and mediated through the enteric nervous system (ENS), which orchestrates the contraction and propulsion of smooth muscle. Gastrointestinal hormone and neurotransmitters serve as key molecular modulators of neurocrine and endocrine crosstalk between CNS and ENS. Gastrointestinal hormone and neurotransmitters primarily include MTL, SP, 5‐HT, SS, and VIP (Zhao et al. 2025). The effects of GP on gastrointestinal hormone regulation, neurotransmitter dynamics in serum were shown in Figure 5A–E. The present findings revealed that loperamide hydrochloride‐induced constipated mice exhibited marked reductions in serum levels of 5‐HT, SP, and MTL compared to the CON group (*p* < 0.05). In contrast, SS levels were significantly elevated with an increase of 16.84 pg/mL (*p* < 0.05). GP intervention reversed these alterations, as evidenced by the GPH group showing elevations of 70.43 ng/mL (5‐HT), 100.40 pg/mL (SP), 84.22 pg/mL (MTL), along with an 11.74 pg/mL reduction in SS levels. VIP exhibits dual functionality, serving both as an inhibitory neuropeptide and a gastrointestinal hormone. As an inhibitory neuropeptide, VIP relaxes gastrointestinal smooth muscle, which can slow intestinal motility. As a gastrointestinal hormone, VIP stimulates chloride and water secretion via the cAMP‐PKA pathway. Homeostatic regulation of this pathway was very important, as functional insufficiency triggered its activation, while overactivation elicited inhibitory feedback, thus insufficient VIP levels will contribute to constipation (Dimidi et al. 2017; Furgała et al. 2023). The results showed that serum VIP levels in the MOD group were significantly lower than those in the CON group, while GP treatment dose‐dependently increased VIP levels in constipated mice (*p* < 0.05). Similar results were also observed in the study of Xie et al. (2024) investigating garlic‐derived fructan/oligofructose mixtures. Zhang, Zu, et al. (2024) demonstrated that the soluble dietary fiber derived from hawthorn alleviated constipation by upregulation of gastrointestinal excitatory hormones such as MTL, GAS, and SP, coupled with concurrent downregulation of inhibitory hormone SS levels (Zhang, Zu, et al. 2024). The results of present study indicated that GP alleviated constipation by modulating serum levels of gastrointestinal hormone regulation, neurotransmitter dynamics related to gut motility, thereby enhancing gastrointestinal peristalsis and promoting digestive mucus secretion.

Immune cytokines play a pivotal role in maintaining intestinal barrier function. Constipation triggers alterations in inflammatory mediators, characterized by elevated levels of pro‐inflammatory cytokines like TNF‐α, IL‐1β, and IL‐17 (Xie et al. 2019). These changes drive inflammatory responses that exacerbate intestinal mucosal injury. To investigate the effects of GP on intestinal immune function in constipated mice, serum inflammatory cytokines were quantified. As shown in Figure 5F–I, increased TNF‐α, IL‐1β, and IL‐17 in MOD group induced mucosal inflammation and compromised the integrity of the intestinal immune barrier. Mechanistically, TNF‐α is recognized as a primary inducer of oxidative and nitrosative stress in inflammatory cells, thereby amplifying intestinal permeability and heightening susceptibility to pathogenic bacterial infections. IL‐10, an anti‐inflammatory cytokine, exerts immunosuppressive effects by inhibiting inflammatory responses. MOD group exhibited significantly elevated serum levels of pro‐inflammatory cytokines and reduced anti‐inflammatory IL‐10 (*p* < 0.05) compared to CON group, demonstrating that constipation‐induced dysregulation of inflammatory cytokines drives inflammatory pathogenesis. GP intervention dose‐dependently reversed these perturbations: GPH intervention reduced the levels of TNF‐α, IL‐1 β, and IL‐17 by 158.83, 33.73, and 14.39 pg/mL respectively, and increased the anti‐inflammatory factor IL‐10 by 9.97 pg/mL. This cytokine rebalancing effect paralleled *Bifidobacterium longum* ‐mediated anti‐inflammatory mechanisms reported by Wang et al. (2022), which significantly reduce the concentration of pro‐inflammatory cytokines TNF‐α and IL‐1 β to improve constipation (Wang et al. 2022). Natural plant polysaccharides have been proven to inhibit inflammation and regulate gut microbiota in intestinal diseases (Lu et al. 2023). Collectively, the present findings revealed that GP as a potent immunoregulator capable of rectifying cytokine homeostasis and mitigating inflammation‐driven constipation pathophysiology, highlighting therapeutic potential through inflammatory pathway modulation.

### 3.6 GP Modulated VIP‐cAMP‐PKA‐AQP3 Signaling Pathway

**FIGURE 6:**
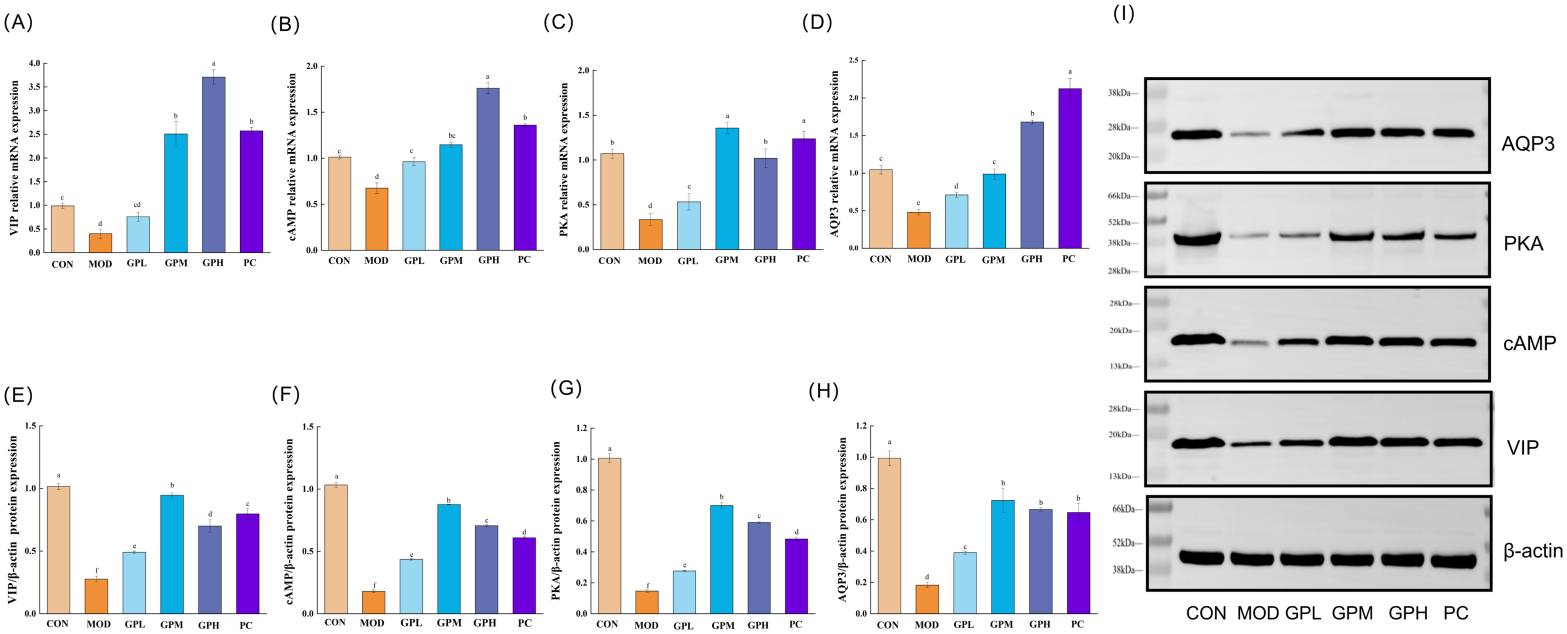
Effects of GP treatment on the mRNA levels (A–D) and the protein expressions (E–I) of VIP‐cAMP‐PKA‐AQP3 in the constipated mice. Data represent the mean ± SEM (*n* = 10). Bars with various lowercase letters differ significantly using one‐way ANOVA followed by the Duncan's test, *p* < 0.05.

The brain‐gut‐microbiome axis has gained substantial recognition as a critical research focus in understanding the pathophysiology of constipation. Within this complex regulatory network, the VIP‐cAMP‐PKA‐AQP3 signaling pathway is as one of the pivotal regulatory mechanisms, orchestrating fluid homeostasis and intestinal motility through coordinated molecular interactions. As shown in Figure 6A–D, compared with the CON group, the MOD group exhibited significant reductions in key mRNA levels along the VIP‐cAMP‐PKA‐AQP3 signaling pathway, and the reductions were observed at 59.60% for VIP (Figure 6A), 33.28% for cAMP (Figure 6B), 68.79% for PKA (Figure 6C), and 59.60% for AQP3 (Figure 6D), respectively (*p* < 0.05). Particularly, both GP and lactulose interventions reversed this trend: the GPH group demonstrated the most pronounced upregulation of VIP and cAMP expression levels, showing 9.27‐ and 2.61‐fold increases relative to the MOD group, respectively. Meanwhile, the medium‐dose GPM group exhibited the highest PKA expression level at 4.06 times that of the MOD group. The lactulose‐treated PC group displayed the most substantial enhancement in AQP3 expression, reaching 4.42‐fold higher levels compared to the MOD group. Consistent with these observations, western blot analysis further validated the expression patterns of key proteins in the VIP‐cAMP‐PKA‐AQP3 signaling pathway within colonic tissues (Figure 6E–I). The results revealed significant downregulation of all investigated proteins in the MOD group relative to CON controls (*p* < 0.05), with respective reductions of 75.22% for VIP, 83.22% for cAMP, 85.42% for PKA, and 80.50% for AQP3. The GPM intervention group demonstrated the most pronounced therapeutic effects, exhibiting 3.42‐, 4.87‐, 4.77‐, and 3.95‐fold increases in VIP, cAMP, PKA, and AQP3 protein expression levels respectively compared to the MOD group. These findings collectively indicated that GP administration effectively upregulated the VIP‐cAMP‐PKA‐AQP3 signaling pathway in the colon of constipated mice. While inhibition of the VIP‐cAMP‐PKA‐AQP3 pathway has been reported to alleviate constipation, our study, alongside other recent evidence, demonstrated that its activation by GP similarly produced a therapeutic effect of constipation. This apparent discrepancy suggested the pathway may function differently depending on the physiological context, which was compensatorily activated when deficient and feedback‐inhibited when overactive. VIP exhibited significant functional diversity: as an inhibitory neuropeptide, it relaxed gastrointestinal smooth muscle, which can decelerate intestinal motility; as a gastrointestinal hormone, it stimulates chloride and water secretion via the cAMP‐PKA pathway. Thus, it was speculated that VIP deficiency was a contributing factor in constipation. Previous study found that VIP levels were reduced in hypothyroid with constipation patients (Mishchuk et al. 2025). The study of Furgała et al. (2023) further demonstrated that patients with constipation‐predominant irritable bowel syndrome exhibited significantly lower VIP levels compared to healthy controls. In this study, GP intervention moderately upregulated VIP expression to a normal physiological level, thereby restoring appropriate intestinal secretory activity and alleviating constipation. Similarly, a garlic fructan/oligofructose mixture has been shown to enhance gut health and relieve constipation by elevating intestinal VIP levels (Xie et al. 2024). Moreover, the downstream cAMP‐PKA pathway acts as a key regulator of intestinal water‐electrolyte balance. In addition, previous study showed that the AQP3 expression at both mRNA and protein levels was decreased in patients with defecation disorder and fluid balance disturbance (Zhu et al. 2023). The GP intervention in present study may alleviate constipation by coordinating these effects to optimize the intraluminal water–electrolyte balance. Activation of the VIPcA‐MP‐PKAAQP3 pathway were also confirmed by which *Lactococcus* alleviated constipation in mice (Tan et al. 2021). Similar findings were observed and supported in several studies. For example, garlic fructans alleviated constipation through upregulation of AQP3 expression (Hu et al. 2025). *Bifidobacterium* and *Lactobacillus* triple viable probiotics similarly alleviated constipation by concurrently upregulating both AQP3 expression and VIP levels (Luo, Xie, et al. 2025). The beneficial effect of ZhiShi DaoZhi decoction on constipation was also correlated with increased AQP3 levels (Fang et al. 2025). The stool‐softening effect of free anthraquinones from *Rheum palmatum* L. in constipated rats involved elevating serum VIP and upregulating the expression of VIP, PKA, and AQP3 (Lv et al. 2024). The role of the VIP‐cAMP‐PKA‐AQP3 pathway in constipation was not unidirectional. The findings suggested that under specific pathophysiological conditions, appropriate activation of this pathway may help restore intestinal water‐metabolic homeostasis, offering a new perspective for precise nutritional intervention in constipation. The studies consistently implicated modulation of VIP‐cAMP‐PKA‐AQP3 pathway as a core mechanism underlying constipation relief.

### 3.7 GP Improved Gut Microbiota in Constipated Mice

**FIGURE 7:**
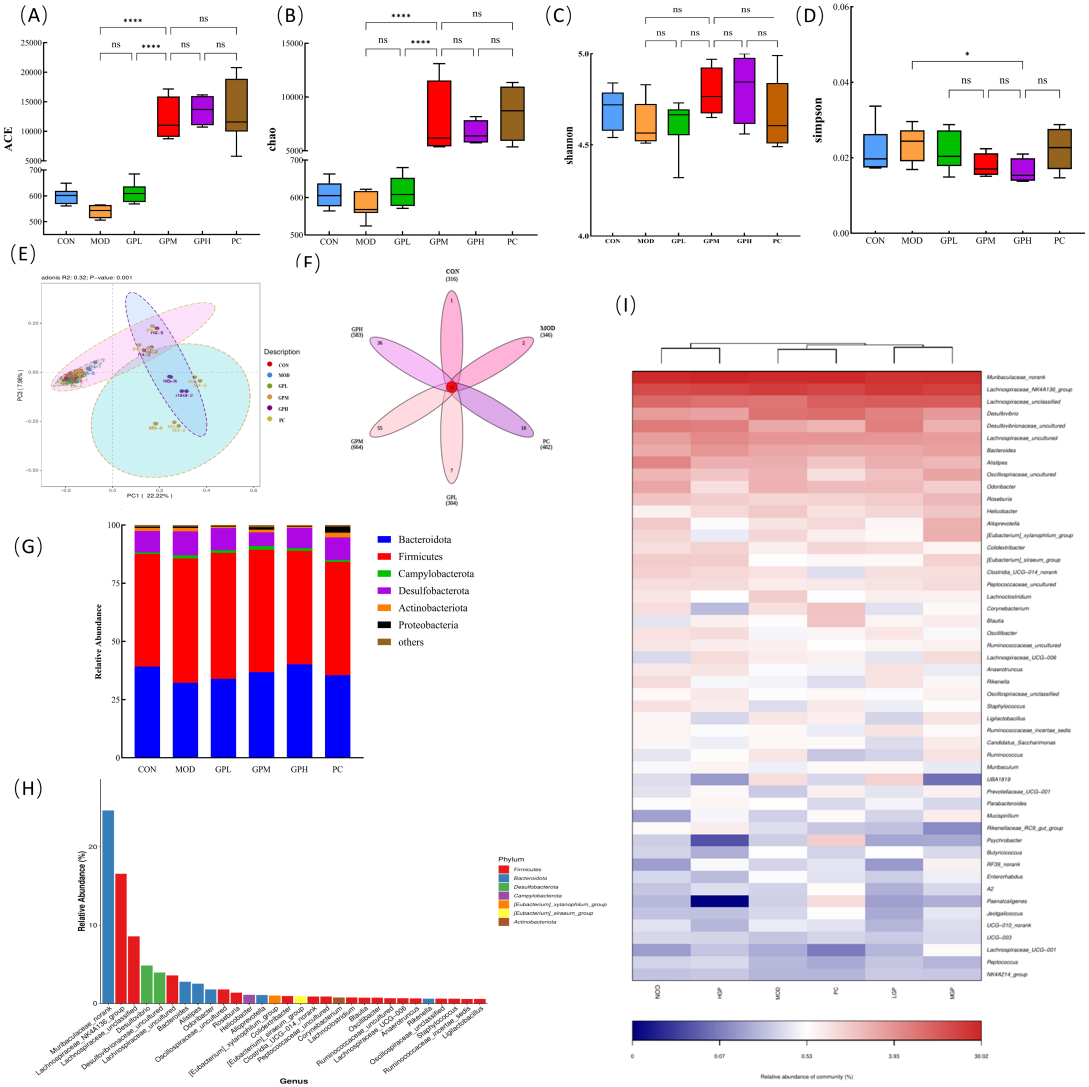
Effects of GP on the gut microbial community composition in mice. (A) α‐diversity ACE index; (B) α‐diversity Chao index; (C) α‐diversity Shannon index; (D) α‐diversity Simpson index; (E) PCA analysis; (F) Venn diagram; (G) relative abundance of the top 7 abundant bacteria at the phylum level; (H) relative abundance of dominant gut microbiota species at the phylum level; (I) heat map analysis of mice intestinal flora at the genus level.

Gut dysbiosis has been recognized as a key contributor to constipation pathogenesis, primarily through its disruption of intestinal motility and SCFAs production, both critical for alleviating constipation symptoms (Lai et al. 2023). To elucidate how GP ameliorates constipation via gut microbiota modulation, the 16S rRNA sequencing on fecal samples from 6 groups was conducted. Alpha diversity indices, reflecting microbial richness (ACE and Chao indices) and diversity (Shannon and Simpson indices), were systematically analyzed (Figure 7A–D). Compared to the CON group, the MOD group exhibited marked reductions in both microbial richness and diversity. The GP intervention obviously reversed this concerning trend, with GPM, GPH, and PC groups showing significant elevations in ACE and Chao indices relative to MOD (*p* < 0.05), indicating enhanced community richness. It is worth noting that the smaller the Simpson index, the higher the species diversity of gut microbiota and the better the health status. The MOD group displayed elevated Simpson index, consistent with constipation‐induced microbial impoverishment. However, GPH intervention significantly reduced Simpson index compared to MOD group (*p* < 0.05), showing the improvement in microbial richness and diversity in GP treatment. These findings collectively highlight GP's capacity to restore gut microbiota homeostasis, likely through structural enrichment and functional activation of SCFA‐producing taxa, thereby addressing constipation at both microbial and physiological levels. Beta diversity is commonly used to compare differences between groups. As shown in Figure 7E, PCA indicated that certain differences exist among different groups, especially in the GPM and GPH groups. A Venn diagram was used to analyze community structure, identifying shared and unique microbial taxa among 6 groups (Figure 7F). Core microbiome analysis revealed 36 conserved OTUs present in all six groups, constituting the foundational microbiota architecture. Notably, medium‐dose GPM and high‐dose GPH interventions demonstrated the highest counts of unique signature. As shown in Figure 7G, *Bacteroidetes* and *Firmicutes* were the predominant phyla, collectively representing the majority of the gut microbiota composition. The *Firmicutes/Bacteroidetes* (F/B) ratio in the CON group was 1.23, while compared to the CON group, the MOD group exhibited a 34.15% increase in the F/B ratio, characterized by elevated *Firmicutes* abundance and reduced Bacteroidetes levels. GP intervention gradually lowering the F/B ratio. Furthermore, the relative abundance of *Desulfovibrio*, a potentially harmful gut bacterium, was significantly reduced following GP treatment, similar result was also found in the study of Liang et al. (2023) (Liang et al. 2023). Guan et al. (2024) also confirmed that plant‐derived polysaccharides ameliorated constipation through prebiotic‐like modulation of gut microbiota. The mechanisms included enriching beneficial bacteria, optimizing the F/B ratio. These microbial shifts improved gut motility and stool properties, and supported the results of present study that GP alleviated functional constipation via microbiota‐directed pathways.

As shown in Figure 7H, the dominant microbial community structure comprised three predominant genera: *Muribaculaceae_norank*, *Lachnospiraceae_NK4A136_group*, and *Lachnospiraceae_unclassified*, belonging to the phyla *Firmicutes* and *Bacteroidetes*, collectively constituting over 56% of the total composition. Nowadays, *Muribaculaceae* has garnered significant attention for its multifaceted role in maintaining host health (Zhu et al. 2024). This bacterial family produces SCFAs, modulates intestinal barrier function and immune responses, and has been recognized as a promising “next‐generation probiotic.” Previous study suggested 3 potential mechanisms through which *Muribaculaceae* may alleviate chronic diseases: enhancing mucin gene expression and secretion to promote epithelial regeneration, thereby mitigating colonic tissue damage and reducing intestinal permeability; metabolizing dietary fibers to generate SCFAs; competing with pathogens for ecological niches and nutrients within the intestinal mucus layer, thereby inhibiting pathogenic bacterial colonization. The *g_Lachnospiraceae_NK4A136_group* was a potential probiotic, which had the potential to correct colonic dysbiosis and restore physiological homeostasis (Tian et al. 2022; Wu et al. 2020). *L*achnospiraceae is involved in maintaining intestinal barrier function and preventing the penetration of harmful substances, thereby reducing the risk of inflammation and disease (Liu et al. 2023).

As illustrated in Figure 7I, genus‐level heatmap analysis revealed significant microbial shifts between different groups. The MOD group exhibited elevated abundances of *Desulfovibrio* and *Helicobacter* compared to the CON group. The abnormal proliferation of *Desulfovibrio* may induce intestinal epithelial damage through hydrogen sulfide production, while increased *Helicobacter* levels are associated with gastrointestinal disorders (Kushkevych et al. 2020). Following GP intervention, beneficial taxa including *Lachnospira* (a butyrate producer) and *Oscillospira* (a fiber‐degrading genus) were obviously enriched, accompanied by reduced *Desulfovibrio* abundance, collectively indicating GP‐mediated restoration of healthier gut microbiota composition.

**FIGURE 8:**
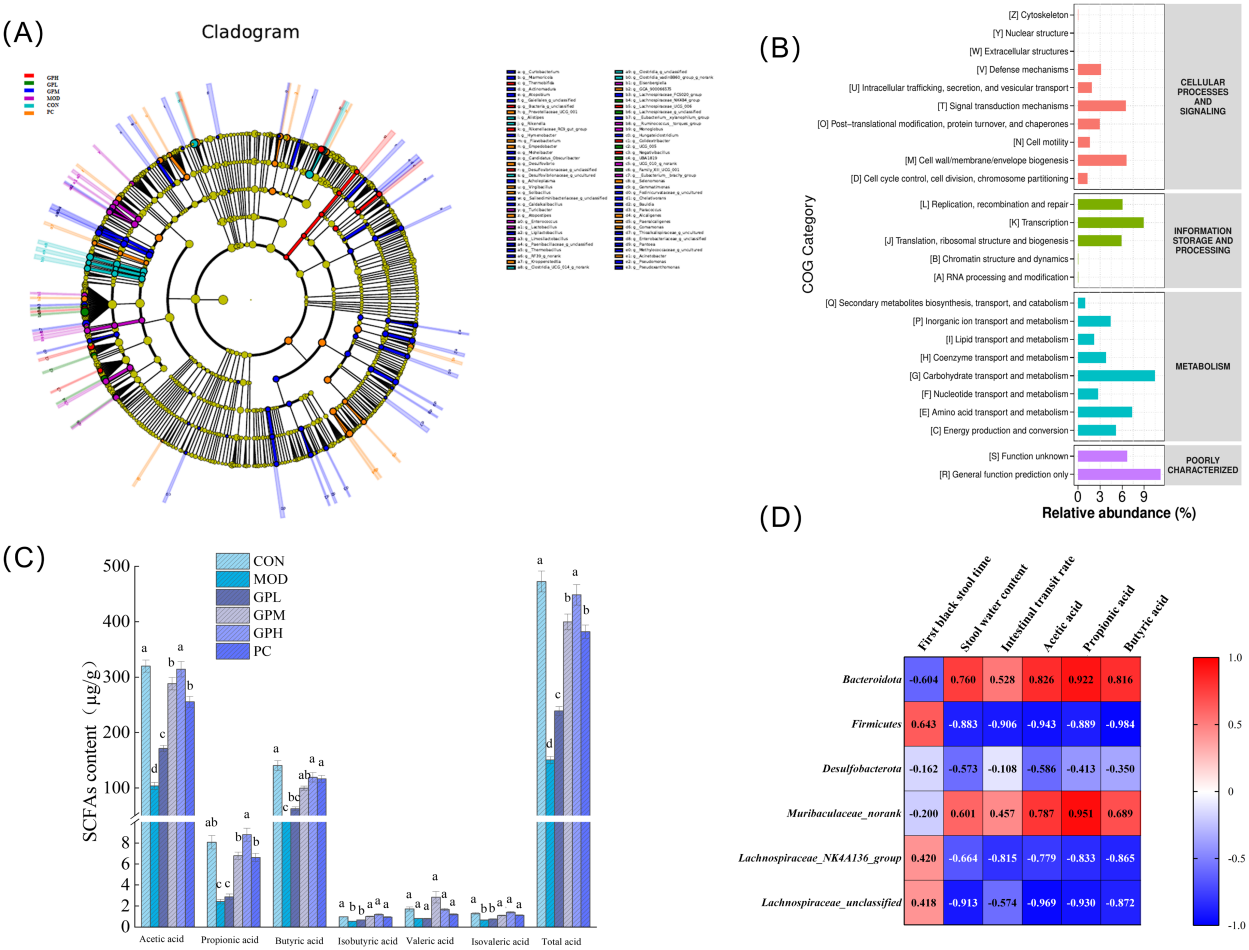
Effects of GP treatment on LEfSe analysis, COG function annotation analysis, correlation analysis, and SCFAs in the fecal contents (μg/g). (A) LEfSe analysis; (B) COG category; (C) the content of SCFAs. (D) Correlation analysis of the bacterial genera and the constipation parameters in mice. Data represent the mean ± SEM (*n* = 10). Bars with various lowercase letters differ significantly using one‐way ANOVA followed by the Duncan's test, *p* < 0.05.

As shown in Figure 8A,B, LEfSe analysis and COG function prediction analyzed the potential mechanism of gut microbiota to alleviate constipation. In Figure 8A, LEfSe shows that *f_Enterobacteriaceae* in MOD group is a dominant family. The *f_Enterobacteriaceae* is a kind of harmful intestinal bacteria, which caused damage to the intestinal micro ecological environment. After GP intervention, *Eubacterium*, *Lachnospiraceae*, *Rikenellaceae* are dominant at the family level (Chen et al. 2021). Among them, *g_Eubacterium_xylanophilum_group* had the ability to decompose oligosaccharides and could decompose acetic acid and butyric acid, which is helpful to maintain intestinal health. The *g_Lachnospiraceae_UCG_006* and *g_Rikenellaceae_RC9_gut_group*, had the ability to promote the decomposition of dietary fiber and produce SCFAs (Guo et al. 2021; Zhou et al. 2021). As shown in Figure 8B, COG functional prediction analysis revealed that GP intervention significantly altered the functional potential of the gut microbiota. Compared to the MOD group, functions related to carbohydrate transport and metabolism were significantly enriched in the GP group. In addition, GP intervention on constipation also affected amino acid transport and metabolism, transcription, signal transduction mechanisms, and cell wall/membrane/envelope biogenesis, etc. These shifts suggested that GP may alleviate constipation by remodeling the gut microbiota, particularly enhancing its capacity for carbohydrate metabolism and SCFAs production, thereby promoting intestinal homeostasis. To conclude, the present findings indicated the positive impacts of GP on constipation were driven by the regulation of gut microbiota and its potential impact on carbohydrate metabolism.

### 3.8 GP Increased SCFAs Production in Constipated Mice

The fecal samples were analyzed for SCFAs content, as shown in Figure 8C. The loperamide hydrochloride‐induced MOD group exhibited significantly lower SCFA levels compared to the CON group. Both GP and lactulose interventions counteracted this decline, with GPM, GPH, and PC groups showing significant increases in total SCFAs levels compared to the MOD group (*p* < 0.05). Comparative analysis revealed superior efficacy of GPH over the PC group, particularly in acetic acid and propionic acid restoration, demonstrating GP effectively regulated SCFAs metabolism. Paticularly, the high‐dose GPH group achieved significantly higher SCFAs concentrations than PC group (*p* < 0.05), highlighting the enhanced capacity of GP to preserve gut microbial metabolic activity in constipation management. The gut microbiota degrades dietary complex carbohydrates to produce SCFAs, which are absorbed by the host and modulate various metabolic processes (Dalile et al. 2019).

Pearson correlation analysis delineated significant microbiota‐physiology relationships in functional constipation pathogenesis, as visualized in Figure 8D. Key constipation parameters including stool water content, intestinal transit rate, and SCFAs (acetic acid, propionic acid, butyric acid) demonstrated positive correlations with the relative abundances of *Bacteroidetes* and *Muribaculaceae_norank*, while exhibiting inverse relationships with *Firmicutes* and *Desulfovibrio* species. Song et al. similarly reported *Muribaculaceae* significantly positively correlated with acetic, propionic, and butyric acid after xylooligosaccharides intervention (Song et al. 2023). The correlation analysis indicated that GP's anti‐constipation effects were closely associated with modulating the abundance of *Bacteroidetes* and *Muribaculaceae*, which were positively correlated with key SCFAs. While a previous study indicated that the well‐established prebiotic polysaccharides, such as inulin, were renowned for its bifidogenic effect, this suggested that GP may engage a partially different microbial network compared to the classic *Bifidobacterium*‐centric modulation by inulin (Qin et al. 2023). GP possessed a robust capacity to stimulate microbial metabolic activity, which is central to constipation relief. The present findings mechanistically explain anti‐constipation effects of GP: by selectively enriching *Bacteroidetes* populations and reducing the F/B ratio, GP modulated microbial‐derived metabolites particularly SCFAs, thus coordinately improved intestinal motility and fluid homeostasis in functional constipation.

## 4 Conclusion

In conclusion, the study demonstrated that GP effectively alleviated loperamide‐induced functional constipation in mice. During the in vitro simulated digestion, GP showed anti‐digestive properties and demonstrated the prebiotic effects of GP in promoting *Bifidobacterium* proliferation. GP exhibited potent dual‐phase antioxidant activity, scavenging ABTS^+^, DPPH•, hydroxyl, and superoxide radicals in vitro while enhancing SOD, GSH‐Px, and CAT activities and reducing MDA in vivo. GP treatment produced dose‐dependent improvements in key constipation parameters in mice. The anti‐constipation mechanism involves multi‐barrier restoration: reinforcement of the mechanical barrier through upregulation of ZO‐1 and occludin mRNA expression, strengthening of the mucus barrier via elevation of mucin gene expression, and stabilization of the immune barrier evidenced by reduced pro‐inflammatory TNF‐α, IL‐1β, and IL‐17 with increased anti‐inflammatory IL‐10. GP further modulated neuroendocrine signaling by elevating excitatory gastrointestinal hormones MTL, GAS, and SP while suppressing inhibitory hormone SS. Moreover, GP intervened in the regulation of the VIP‐cAMP‐PKA‐AQP3 pathway, improved intestinal water transport, increased mucus secretion, protected the stability of the intestinal barrier and water balance, ultimately ameliorating constipation. Critically, GP ameliorated constipation by remodeling the gut microbiota: it selectively enriched *Bacteroidetes*, reduced the F/B ratio, and consequently modulated microbial‐derived metabolites, particularly enhancing SCFAs production. These coordinated actions on barrier function, VIP pathway, and the microbiota‐SCFAs axis improved intestinal homeostasis, underpinning the efficacy against functional constipation of GP. Therefore, with its multifaceted anti‐constipation mechanisms, GP emerges as a promising natural bioactive ingredient for functional foods targeting intestinal health enhancement.

## Author Contributions

**Jingfang Li:** conceptualization, formal analysis, data curation, writing original draft preparation, funding acquisition, writing review and editing. **Jiaxin Miao:** methodology, data curation. **Tianyi Li:** methodology, data curation. **Chanyuan Xie:** visualization. **Wentao Xu:** supervision, writing review and editing. **Shimin Chang:** supervision. **Ran Chai:** methodology, funding acquisition.

## Conflicts of Interest

The authors declare no conflicts of interest.

## Supporting information


**Figure S1:** The main structure of garlic polysaccharide.
**Table S1:** Primer information.
**Table S2:** Changes of reducing sugar contents of GP.

## Data Availability

The data that support the findings of this study are available from the corresponding author upon reasonable request.

## References

[fsn371659-bib-0001] Bai, J. , Y. Cai , Z. Huang , et al. 2022. “Shouhui Tongbian Capsule Ameliorates Constipation via Gut Microbiota‐5‐HT‐Intestinal Motility Axis.” Biomedicine & Pharmacotherapy 154: 113627. 36058152 10.1016/j.biopha.2022.113627

[fsn371659-bib-0002] Barberio, B. , C. Judge , E. V. Savarino , and A. C. Ford . 2021. “Global Prevalence of Functional Constipation According to the Rome Criteria: A Systematic Review and Meta‐Analysis.” Lancet Gastroenterology & Hepatology 6, no. 8: 638–648. 34090581 10.1016/S2468-1253(21)00111-4

[fsn371659-bib-0003] Brodkorb, A. , L. Egger , M. Alminger , et al. 2019. “INFOGEST Static In Vitro Simulation of Gastrointestinal Food Digestion.” Nature Protocols 14, no. 4: 991–1014. 30886367 10.1038/s41596-018-0119-1

[fsn371659-bib-0004] Camilleri, M. 2019. “Leaky Gut: Mechanisms, Measurement and Clinical Implications in Humans.” Gut 68, no. 8: 1516–1526. 31076401 10.1136/gutjnl-2019-318427 PMC6790068

[fsn371659-bib-0005] Chen, X. , B. Cai , J. Wang , et al. 2021. “Mulberry Leaf‐Derived Polysaccharide Modulates the Immune Response and Gut Microbiota Composition in Immunosuppressed Mice.” Journal of Functional Foods 83: 104545.

[fsn371659-bib-0006] Dalile, B. , L. Van Oudenhove , B. Vervliet , and K. Verbeke . 2019. “The Role of Short‐Chain Fatty Acids in Microbiota–Gut–Brain Communication.” Nature Reviews Gastroenterology & Hepatology 16, no. 8: 461–478. 31123355 10.1038/s41575-019-0157-3

[fsn371659-bib-0007] Deng, M. , J. Ye , R. Zhang , et al. 2024. “Shatianyu (*Citrus grandis* l. Osbeck) Whole Fruit Alleviated Loperamide‐Induced Constipation via Enhancing Gut Microbiota‐Mediated Intestinal Serotonin Secretion and Mucosal Barrier Homeostasis.” Food & Function 15, no. 21: 10614–10627. 39373198 10.1039/d4fo02765e

[fsn371659-bib-0008] Dimidi, E. , S. Christodoulides , S. M. Scott , and K. Whelan . 2017. “Mechanisms of Action of Probiotics and the Gastrointestinal Microbiota on Gut Motility and Constipation.” Advances in Nutrition 8, no. 3: 484–494. 28507013 10.3945/an.116.014407 PMC5421123

[fsn371659-bib-0009] Fang, L. , X. Yi , J. Shen , N. Deng , and X. Peng . 2025. “Gut‐Brain Axis Mediated by Intestinal Content Microbiota Was Associated With Zhishi Daozhi Decoction on Constipation.” Frontiers in Cellular and Infection Microbiology 15: 1539277. 39963403 10.3389/fcimb.2025.1539277 PMC11830728

[fsn371659-bib-0010] Fernández‐García, P. , S. Taxerås , M. Reyes‐Farias , et al. 2024. “Claudin‐1 as a Novel Target Gene Induced in Obesity and Associated to Inflammation, Fibrosis, and Cell Differentiation.” European Journal of Endocrinology 190, no. 3: 201–210. 38375549 10.1093/ejendo/lvae018

[fsn371659-bib-0011] Filippone, A. , A. Ardizzone , V. Bova , et al. 2022. “A Combination of Xyloglucan, Pea Protein and Chia Seed Ameliorates Intestinal Barrier Integrity and Mucosa Functionality in a Rat Model of Constipation‐Predominant Irritable Bowel Syndrome.” Journal of Clinical Medicine 11, no. 23: 7073. 36498647 10.3390/jcm11237073 PMC9739531

[fsn371659-bib-0012] Fu, C. , K. Ye , S. Ma , et al. 2023. “Simulated Gastrointestinal Digestion and Gut Microbiota Fermentation of Polysaccharides From Agaricus Bisporus.” Food Chemistry 418: 135849. 36963137 10.1016/j.foodchem.2023.135849

[fsn371659-bib-0013] Furgała, A. , K. Ciesielczyk , M. Przybylska‐Feluś , K. Jabłoński , K. Gil , and M. Zwolińska‐Wcisło . 2023. “Postprandial Effect of Gastrointestinal Hormones and Gastric Activity in Patients With Irritable Bowel Syndrome.” Scientific Reports 13, no. 1: 9420. 37296188 10.1038/s41598-023-36445-1 PMC10256731

[fsn371659-bib-0014] Gou, H. , Y. Zhang , L. Ren , Z. Li , and L. Zhang . 2022. “How Do Intestinal Probiotics Restore the Intestinal Barrier?” Frontiers in Microbiology 13: 929346. 35910620 10.3389/fmicb.2022.929346 PMC9330398

[fsn371659-bib-0015] Grondin, J. A. , Y. H. Kwon , P. M. Far , S. Haq , and W. I. Khan . 2020. “Mucins in Intestinal Mucosal Defense and Inflammation: Learning From Clinical and Experimental Studies.” Frontiers in Immunology 11: 2054. 33013869 10.3389/fimmu.2020.02054 PMC7500085

[fsn371659-bib-0016] Gu, W. , Y. Wang , L. Zeng , et al. 2020. “Polysaccharides From Polygonatum Kingianum Improve Glucose and Lipid Metabolism in Rats Fed a High Fat Diet.” Biomedicine & Pharmacotherapy 125: 109910. 32028238 10.1016/j.biopha.2020.109910

[fsn371659-bib-0017] Guan, C. , B. Zhang , Y. Wang , et al. 2024. “Effects of Wheat Germ Polysaccharides Prepared by Ultra‐High Pressure on Functional Constipation and Gut Microbiota.” Food Bioscience 57: 103347.

[fsn371659-bib-0018] Gulcin, İ. 2020. “Antioxidants and Antioxidant Methods: An Updated Overview.” Archives of Toxicology 94, no. 3: 651–715. 32180036 10.1007/s00204-020-02689-3

[fsn371659-bib-0019] Guo, W. , Q. Xiang , B. Mao , et al. 2021. “Protective Effects of Microbiome‐Derived Inosine on Lipopolysaccharide‐Induced Acute Liver Damage and Inflammation in Mice via Mediating the TLR4/NF‐κb Pathway.” Journal of Agricultural and Food Chemistry 69, no. 27: 7619–7628. 34156842 10.1021/acs.jafc.1c01781

[fsn371659-bib-0020] Hao, W. , R. Cha , M. Wang , et al. 2023. “Ligand‐Modified Gold Nanoparticles as Mitochondrial Modulators: Regulation of Intestinal Barrier and Therapy for Constipation.” ACS Nano 17, no. 14: 13377–13392. 37449942 10.1021/acsnano.3c01656

[fsn371659-bib-0021] He, J. , Y. Zhang , M. Li , et al. 2025. “Polysaccharide Extracted From Peony Seed Meal Preventive Effect of on Loperamide‐Induced Constipation in Rats.” International Journal of Biological Macromolecules 308: 142391. 40139599 10.1016/j.ijbiomac.2025.142391

[fsn371659-bib-0022] Hossen, I. , W. Hua , L. Ting , et al. 2022. “Phytochemicals and Inflammatory Bowel Disease: A Review.” Critical Reviews in Food Science and Nutrition 60, no. 8: 1321–1345. 10.1080/10408398.2019.1570913 30729797

[fsn371659-bib-0023] Hu, Y. , X. Bai , L. Li , et al. 2025. “Anticonstipation Activity of Garlic Fructans in Loperamide‐Induced Constipated Mice: Impact of Polymerization Degree.” Journal of Agricultural and Food Chemistry 73, no. 12: 7310–7324. 40098447 10.1021/acs.jafc.4c12689

[fsn371659-bib-0024] Huo, J. , L. Wu , J. Lv , H. Cao , and Q. Gao . 2022. “Effect of Fruit Intake on Functional Constipation: A Systematic Review and Meta‐Analysis of Randomized and Crossover Studies.” Frontiers in Nutrition 9: 1018502. 36276840 10.3389/fnut.2022.1018502 PMC9583540

[fsn371659-bib-0025] Jiang, H. , R. Ma , F. Ji , et al. 2024. “Structure Characterization of Polysaccharides From Cistanche Deserticola and Their Neuroprotective Effects Against Oxidative Stress in Slow Transit Constipation Mice.” International Journal of Biological Macromolecules 260: 129527. 38246435 10.1016/j.ijbiomac.2024.129527

[fsn371659-bib-0026] Jie, Z. , J. Liu , M. Shu , Y. Ying , and H. Yang . 2022. “Detection Strategies for Superoxide Anion: A Review.” Talanta 236: 122892. 34635271 10.1016/j.talanta.2021.122892

[fsn371659-bib-0027] Keung, W. , W. Zhang , H. Luo , K. Chan , Y. Chan , and J. Xu . 2025. “Correlation Between the Structures of Natural Polysaccharides and Their Properties in Regulating Gut Microbiota: Current Understanding and Beyond.” Carbohydrate Polymers 352: 123209. 39843110 10.1016/j.carbpol.2024.123209

[fsn371659-bib-0028] Kim, H. , E. Jeong , C. Park , et al. 2023. “Modulation of Gut Microbiota Ecosystem by a Glucan‐Rich Snail Mucin Heteropolysaccharide Attenuates Loperamide‐Induced Constipation.” International Journal of Biological Macromolecules 253: 126560. 37640190 10.1016/j.ijbiomac.2023.126560

[fsn371659-bib-0029] Kruk, J. , H. Y. Aboul‐Enein , A. Kładna , and J. E. Bowser . 2019. “Oxidative Stress in Biological Systems and Its Relation With Pathophysiological Functions: The Effect of Physical Activity on Cellular Redox Homeostasis.” Free Radical Research 53, no. 5: 497–521. 31039624 10.1080/10715762.2019.1612059

[fsn371659-bib-0030] Kurashima, Y. , and H. Kiyono . 2017. “Mucosal Ecological Network of Epithelium and Immune Cells for Gut Homeostasis and Tissue Healing.” Annual Review of Immunology 35, no. 1: 119–147. 10.1146/annurev-immunol-051116-052424 28125357

[fsn371659-bib-0031] Kushkevych, I. , J. Cejnar , J. Treml , D. Dordević , P. Kollar , and M. Vítězová . 2020. “Recent Advances in Metabolic Pathways of Sulfate Reduction in Intestinal Bacteria.” Cells 9, no. 3: 698. 32178484 10.3390/cells9030698 PMC7140700

[fsn371659-bib-0032] Lai, H. , Y. Li , Y. He , et al. 2023. “Effects of Dietary Fibers or Probiotics on Functional Constipation Symptoms and Roles of Gut Microbiota: A Double‐Blinded Randomized Placebo Trial.” Gut Microbes 15, no. 1: 2197837. 37078654 10.1080/19490976.2023.2197837 PMC10120550

[fsn371659-bib-0033] Li, J. , Y. Qin , X. Yu , et al. 2019. “In Vitro Simulated Digestion and In Vivo Metabolism of Chlorogenic Acid Dimer From Gynura Procumbens (Lour.) Merr.: Enhanced Antioxidant Activity and Different Metabolites of Blood and Urine.” Journal of Food Biochemistry 43, no. 6: e12654. 31353620 10.1111/jfbc.12654

[fsn371659-bib-0034] Li, R. , S. Xu , B. Li , et al. 2023. “Gut Indigenous *ruminococcus gnavus* Alleviates Constipation and Stress‐Related Behaviors in Mice With Loperamide‐Induced Constipation.” Food & Function 14, no. 12: 5702–5715. 37278206 10.1039/d2fo03574j

[fsn371659-bib-0035] Li, S. , G. Li , D. Liu , et al. 2024. “Peach Gum Polysaccharide Protects Intestinal Barrier Function, Reduces Inflammation and Oxidative Stress, and Alleviates Pulmonary Inflammation Induced by *enterococcus faecium* .” Journal of Functional Foods 115: 106098.

[fsn371659-bib-0036] Liang, Y. , X. Wei , J. Deng , et al. 2023. “Integrating Omics and Network Pharmacology Reveals the Anti‐Constipation Role of Chitosan With Different Molecular Weights in Constipated Mice.” International Journal of Biological Macromolecules 235: 123930. 36889616 10.1016/j.ijbiomac.2023.123930

[fsn371659-bib-0037] Liu, C. , P. Du , Y. Cheng , et al. 2021. “Study on Fecal Fermentation Characteristics of Aloe Polysaccharides In Vitro and Their Predictive Modeling.” Carbohydrate Polymers 256: 117571. 33483068 10.1016/j.carbpol.2020.117571

[fsn371659-bib-0083] Liu, W. , L. Yu , Q. Chen , et al. 2025. “Poria Cocos Polysaccharides Alleviate Obesity‐related Adipose Tissue Insulin Resistance via Gut Microbiota‐derived Short‐chain Fatty Acids Activation of FGF21/PI3K/AKT Signaling.” Food Research International 215: 116671. 40484558 10.1016/j.foodres.2025.116671

[fsn371659-bib-0038] Liu, X. , C. Jian , M. Li , F. Wei , H. Liu , and X. Qin . 2022. “Microbiome‐Metabolomics Deciphers the Effects of *Cistanche deserticola* Polysaccharides on Aged Constipated Rats.” Food & Function 13, no. 7: 3993–4008. 35315484 10.1039/d2fo00008c

[fsn371659-bib-0039] Liu, Z. , S. Fayyaz , D. Zhao , et al. 2023. “Polygonatum Sibiricum Polysaccharides Improve Cognitive Function in d‐Galactose‐Induced Aging Mice by Regulating the Microbiota‐Gut‐Brain Axis.” Journal of Functional Foods 103: 105476.

[fsn371659-bib-0040] Lou, J. , B. Zhang , Y. Zheng , M. Liu , and Y. Qu . 2024. “Hawthorn Pectin Plays a Protective Role in Myocardial Ischaemia by Regulating Intestinal Flora and Short Chain Fatty Acids.” Current Research in Food Science 9: 100863. 39416365 10.1016/j.crfs.2024.100863 PMC11480239

[fsn371659-bib-0041] Lu, H. , M. Shen , Y. Chen , Q. Yu , T. Chen , and J. Xie . 2023. “Alleviative Effects of Natural Plant Polysaccharides Against DSS‐Induced Ulcerative Colitis via Inhibiting Inflammation and Modulating Gut Microbiota.” Food Research International 167: 112630. 37087227 10.1016/j.foodres.2023.112630

[fsn371659-bib-0042] Lu, Y. , X. Zhou , Y. Wu , et al. 2024. “Metabolites 13, 14‐Dihydro‐15‐Keto‐PGE2 Participates in *Bifidobacterium animalis* f1‐7 to Alleviate Opioid‐Induced Constipation by 5‐HT Pathway.” Molecular Nutrition & Food Research 68, no. 3: 2200846. 10.1002/mnfr.202200846 38054625

[fsn371659-bib-0043] Luo, M. , P. Xie , X. Deng , J. Fan , and L. Xiong . 2025. “Bifidobacterium Lactobacillus Triple Viable Alleviates Slow Transit Constipation by Regulating Gut Microbiota and Metabolism.” Journal of Gastroenterology and Hepatology 40, no. 6: 16960. 10.1111/jgh.16960 PMC12136808 40183209

[fsn371659-bib-0044] Luo, Y. , R. Tang , and Y. Huang . 2025. “Differences in Structure, Antioxidant Capacity and Gut Microbiota Modulation of Red Raspberry Pectic Polysaccharides Extracted by Different Methods.” Food Research International 211: 116474. 40356136 10.1016/j.foodres.2025.116474

[fsn371659-bib-0045] Lv, H. , J. Niu , W. Pan , et al. 2024. “Stool‐Softening Effect and Action Mechanism of Free Anthraquinones Extracted From *Rheum palmatum* l. On Water Deficit‐Induced Constipation in Rats.” Journal of Ethnopharmacology 319: 117336. 37907143 10.1016/j.jep.2023.117336

[fsn371659-bib-0046] Mishchuk, V. , H. Kozinchuk , O. Venhrovych , U. Shalamai , and T. Salyzhyn . 2025. “The Level of Vasoactive Intestinal Polypeptide in Patients With Hypothyroidism and Impaired Intestinal Function, and Its Association With Markers of Visceral Hypersensitivity.” International Journal of Endocrinology 21, no. 4: 395–400.

[fsn371659-bib-0047] Pacholczyk‐Sienicka, B. , J. Modranka , and G. Ciepielowski . 2024. “Comparative Analysis of Bioactive Compounds in Garlic Owing to the Cultivar and Origin.” Food Chemistry 439: 138141. 38061302 10.1016/j.foodchem.2023.138141

[fsn371659-bib-0048] Premarathna, A. D. , T. A. Ahmed , G. Kulshreshtha , et al. 2024. “Polysaccharides From Red Seaweeds: Effect of Extraction Methods on Physicochemical Characteristics and Antioxidant Activities.” Food Hydrocolloids 147: 109307.

[fsn371659-bib-0049] Qin, Y. , L. Wang , X. Yang , et al. 2023. “Inulin: Properties and Health Benefits.” Food & Function 14, no. 7: 2948–2968. 36876591 10.1039/d2fo01096h

[fsn371659-bib-0050] Ren, X. , L. Liu , Y. Gamallat , B. Zhang , and Y. Xin . 2017. “Enteromorpha and Polysaccharides From Enteromorpha Ameliorate Loperamide‐Induced Constipation in Mice.” Biomedicine & Pharmacotherapy 96: 1075–1081. 29198923 10.1016/j.biopha.2017.11.119

[fsn371659-bib-0051] Ritota, M. , L. Casciani , B. Han , et al. 2012. “Traceability of Italian Garlic (*Allium sativum* l.) by Means of HRMAS‐NMR Spectroscopy and Multivariate Data Analysis.” Food Chemistry 135, no. 2: 684–693. 22868146 10.1016/j.foodchem.2012.05.032

[fsn371659-bib-0052] Schwayer, C. , S. Shamipour , K. Pranjic‐Ferscha , et al. 2019. “Mechanosensation of Tight Junctions Depends on ZO‐1 Phase Separation and Flow.” Cell 179, no. 4: 937–952. 31675500 10.1016/j.cell.2019.10.006

[fsn371659-bib-0053] Senapati, S. , S. Das , and S. K. Batra . 2010. “Mucin‐Interacting Proteins: From Function to Therapeutics.” Trends in Biochemical Sciences 35, no. 4: 236–245. 19913432 10.1016/j.tibs.2009.10.003 PMC3030310

[fsn371659-bib-0054] Shao, X. , J. Li , H. Zhang , et al. 2023. “Anti‐Inflammatory Effects and Molecular Mechanisms of Bioactive Small Molecule Garlic Polysaccharide.” Frontiers in Nutrition 9: 1092873. 36698476 10.3389/fnut.2022.1092873 PMC9868249

[fsn371659-bib-0055] Shi, L. , L. Shi , M. Wei , et al. 2024. “Prevalence, Risk Factors, Impact and Management of Constipation Among Adults in Urumqi, China: A Cross‐Sectional Survey.” Frontiers in Nutrition 11: 1451527. 39555186 10.3389/fnut.2024.1451527 PMC11563834

[fsn371659-bib-0056] Shon, D. J. , D. Fernandez , N. M. Riley , M. J. Ferracane , and C. R. Bertozzi . 2022. “Structure‐Guided Mutagenesis of a Mucin‐Selective Metalloprotease From *Akkermansia muciniphila* Alters Substrate Preferences.” Journal of Biological Chemistry 298, no. 5: 101917. 35405095 10.1016/j.jbc.2022.101917 PMC9118916

[fsn371659-bib-0057] Song, H. , R. Guo , X. Sun , et al. 2023. “Xylooligosaccharides From Corn Cobs Alleviate Loperamide‐Induced Constipation in Mice via Modulation of Gut Microbiota and SCFA Metabolism.” Food & Function 14, no. 19: 8734–8746. 37694718 10.1039/d3fo02688d

[fsn371659-bib-0058] Tan, Q. , J. Hu , Y. Zhou , et al. 2021. “Inhibitory Effect of *Lactococcus lactis* Subsp. Lactis HFY14 on Diphenoxylate‐Induced Constipation in Mice by Regulating the VIP‐cAMP‐PKA‐AQP3 Signaling Pathway.” Drug Design, Development and Therapy 15: 1971–1980. 34007157 10.2147/DDDT.S309675 PMC8123977

[fsn371659-bib-0059] Tian, H. , Z. Wen , Z. Liu , Y. Guo , G. Liu , and B. Sun . 2022. “Comprehensive Analysis of Microbiome, Metabolome and Transcriptome Revealed the Mechanisms of *Moringa oleifera* Polysaccharide on Preventing Ulcerative Colitis.” International Journal of Biological Macromolecules 222: 573–586. 36115453 10.1016/j.ijbiomac.2022.09.100

[fsn371659-bib-0060] Vriesman, M. H. , I. J. Koppen , M. Camilleri , C. Di Lorenzo , and M. A. Benninga . 2020. “Management of Functional Constipation in Children and Adults.” Nature Reviews Gastroenterology & Hepatology 17, no. 1: 21–39. 31690829 10.1038/s41575-019-0222-y

[fsn371659-bib-0061] Wald, A. 2016. “Constipation: Advances in Diagnosis and Treatment.” JAMA 315, no. 2: 185–191. 26757467 10.1001/jama.2015.16994

[fsn371659-bib-0062] Wang, L. , M. Chai , J. Wang , et al. 2022. “ *Bifidobacterium longum* Relieves Constipation by Regulating the Intestinal Barrier of Mice.” Food & Function 13, no. 9: 5037–5049. 35394000 10.1039/d1fo04151g

[fsn371659-bib-0063] Wang, X. , X. Li , L. Zhang , et al. 2024. “Recent Progress in Plant‐Derived Polysaccharides With Prebiotic Potential for Intestinal Health by Targeting Gut Microbiota: A Review.” Critical Reviews in Food Science and Nutrition 64, no. 33: 12242–12271. 37651130 10.1080/10408398.2023.2248631

[fsn371659-bib-0064] Wells, J. M. , Y. Gao , N. de Groot , M. M. Vonk , L. Ulfman , and R. J. van Neerven . 2022. “Babies, Bugs, and Barriers: Dietary Modulation of Intestinal Barrier Function in Early Life.” Annual Review of Nutrition 42, no. 1: 165–200. 10.1146/annurev-nutr-122221-103916 35697048

[fsn371659-bib-0065] Wu, D. , Y. Fu , H. Guo , et al. 2021. “In Vitro Simulated Digestion and Fecal Fermentation of Polysaccharides From Loquat Leaves: Dynamic Changes in Physicochemical Properties and Impacts on Human Gut Microbiota.” International Journal of Biological Macromolecules 168: 733–742. 33232697 10.1016/j.ijbiomac.2020.11.130

[fsn371659-bib-0066] Wu, D. , W. Liu , Q. Yuan , et al. 2022. “Dynamic Variations in Physicochemical Characteristics of Oolong Tea Polysaccharides During Simulated Digestion and Fecal Fermentation In Vitro.” Food Chemistry: X 14: 100288. 35342881 10.1016/j.fochx.2022.100288 PMC8942832

[fsn371659-bib-0067] Wu, J. , G. Yu , X. Zhang , et al. 2024. “A Fructan‐Type Garlic Polysaccharide Upregulates Immune Responses in Macrophage Cells and in Immunosuppressive Mice.” Carbohydrate Polymers 344: 122530. 39218552 10.1016/j.carbpol.2024.122530

[fsn371659-bib-0068] Wu, M. , T. Chou , C. Huang , and J. Hsiao . 2020. “A Potential Probiotic‐Lachnospiraceae NK4a136 Group: Evidence From the Restoration of the Dietary Pattern From a High‐Fat Diet.” Research Square 10: 1–24.

[fsn371659-bib-0069] Xie, C. , W. Gao , X. Li , S. Luo , D. Wu , and F. Y. Chye . 2023. “Garlic ( *Allium sativum* l.) Polysaccharide Ameliorates Type 2 Diabetes Mellitus (t2DM) via the Regulation of Hepatic Glycogen Metabolism.” NFS Journal 31: 19–27.

[fsn371659-bib-0070] Xie, C. , W. Gao , X. Liang , and F. Y. Chye . 2024. “Effects of Garlic‐Derived Fructan and Oligofructose Mixtures on Intestinal Health and Constipation Relief in Mice.” Journal of the Science of Food and Agriculture 104, no. 12: 7476–7487. 38742546 10.1002/jsfa.13567

[fsn371659-bib-0071] Xie, S. , B. Liu , H. Ye , et al. 2019. “Dendrobium Huoshanense Polysaccharide Regionally Regulates Intestinal Mucosal Barrier Function and Intestinal Microbiota in Mice.” Carbohydrate Polymers 206: 149–162. 30553308 10.1016/j.carbpol.2018.11.002

[fsn371659-bib-0072] Xu, J. , Z. Weng , Q. Cui , et al. 2025. “Current Research on the Regulation of Glycolipid Metabolism by Plant‐Derived Active Polysaccharides.” Trends in Food Science & Technology 159: 104959.

[fsn371659-bib-0073] Yao, D. , M. Wu , Y. Dong , et al. 2022. “In Vitro Fermentation of Fructooligosaccharide and Galactooligosaccharide and Their Effects on Gut Microbiota and SCFAs in Infants.” Journal of Functional Foods 99: 105329.

[fsn371659-bib-0074] Zeisel, M. B. , P. Dhawan , and T. F. Baumert . 2019. “Tight Junction Proteins in Gastrointestinal and Liver Disease.” Gut 68, no. 3: 547–561. 30297438 10.1136/gutjnl-2018-316906 PMC6453741

[fsn371659-bib-0075] Zhan, X. , W. Peng , Z. Wang , et al. 2022. “Polysaccharides From Garlic Protect Against Liver Injury in DSS‐Induced Inflammatory Bowel Disease of Mice via Suppressing Pyroptosis and Oxidative Damage.” Oxidative Medicine and Cellular Longevity 2022, no. 1: 2042163. 36017235 10.1155/2022/2042163 PMC9398839

[fsn371659-bib-0076] Zhang, H. , Q. Zu , J. Zhang , et al. 2024. “Soluble Dietary Fiber of Hawthorn Relieves Constipation Induced by Loperamide Hydrochloride by Improving Intestinal Flora and Inflammation, Thereby Regulating the Aquaporin Ion Pathway in Mice.” Food 13, no. 14: 2220. 10.3390/foods13142220 PMC11275587 39063304

[fsn371659-bib-0077] Zhang, Y. , L. Li , X. Ma , et al. 2024. “Extraction, Purification, Structural Features, Modifications, Bioactivities, Structure–Activity Relationships, and Applications of Polysaccharides From Garlic: A Review.” International Journal of Biological Macromolecules 265: 131165. 38547941 10.1016/j.ijbiomac.2024.131165

[fsn371659-bib-0078] Zhao, X. , Y. Qiu , L. Liang , and X. Fu . 2025. “Interkingdom Signaling Between Gastrointestinal Hormones and the Gut Microbiome.” Gut Microbes 17, no. 1: 2456592. 39851261 10.1080/19490976.2025.2456592 PMC11776477

[fsn371659-bib-0079] Zhou, J. , H. Zhang , S. Wu , et al. 2021. “Supplemental Xylooligosaccharide Modulates Intestinal Mucosal Barrier and Cecal Microbiota in Laying Hens Fed Oxidized Fish Oil.” Frontiers in Microbiology 12: 635333. 33692770 10.3389/fmicb.2021.635333 PMC7937631

[fsn371659-bib-0080] Zhu, C. , X. Nie , Q. Lu , Y. Bai , and Z. Jiang . 2023. “Roles and Regulation of Aquaporin‐3 in Maintaining the Gut Health: An Updated Review.” Frontiers in Physiology 14: 1264570. 38089478 10.3389/fphys.2023.1264570 PMC10714013

[fsn371659-bib-0081] Zhu, Y. , B. Chen , X. Zhang , et al. 2024. “Exploration of the Muribaculaceae Family in the Gut Microbiota: Diversity, Metabolism, and Function.” Nutrients 16, no. 16: 2660. 39203797 10.3390/nu16162660 PMC11356848

[fsn371659-bib-0082] Zouali, M. 2021. “B Lymphocytes, the Gastrointestinal Tract and Autoimmunity.” Autoimmunity Reviews 20, no. 4: 102777. 33609796 10.1016/j.autrev.2021.102777

