# Supplementary material for: The Anti‐Constipation Effect of Garlic Polysaccharides: Roles of Gut Barrier Integrity, VIP Pathway, and the Microbiota‐SCFAs Axis
Source: Food Sci Nutr. 2026 Mar 17;14(3):e71659. doi: 10.1002/fsn3.71659 (PMC13093614; doi:10.1002/fsn3.71659)
Supplement: Supplementary file 1 — Figure S1: The main structure of garlic polysaccharide. Table S1: Primer information. Table S2: Changes of reducing sugar contents of GP. [file FSN3-14-e71659-s001.doc]

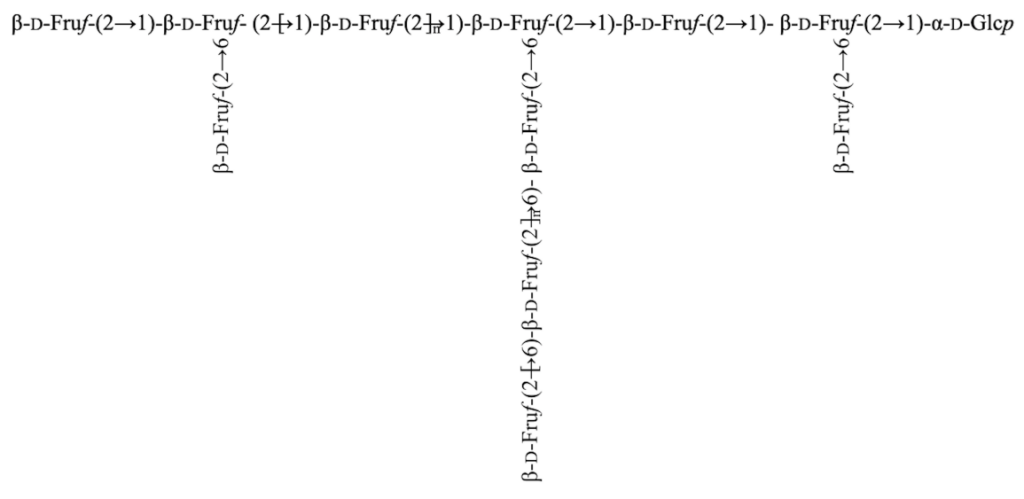
 **Supplementary Figure 1**

Fig S1. The main structure of garlic polysaccharide

**Supplementary Table 1**

Table S1. Primer information

| Primer Information | Target genes | Primers（5’-3’） |
| --- | --- | --- |
| NM_008084.2 | M-GAPDH-S | CCTCGTCCCGTAGACAAAATG |
|  | M-GAPDH-A | TGAGGTCAATGAAGGGGTCGT |
| NM_009386.2 | M-ZO1-S | GGGAAAACCCGAAACTGATG |
|  | M-ZO1-A | GCTGTACTGTGAGGGCAACG |
| NM_016674.4 | M-Claudin-1-S | GTGTCCTACTTTCCTGCTCCTGT |
|  | M-Claudin-1-A | TCACACATAGTCTTTCCCACTAGAAG |
| NM_023566.4 | M- MUC2-S | GAAGCCAGATCCCGAAACCA |
|  | M- MUC2-A | GAATCGGTAGACATCGCCGT |
| NM_001360536.1 | M-Occludin-S | CACCTCCTTACAGACCTGATGAAT |
|  | M-Occludin-A | AGCCACCTCCGTAGCCAAA |
| NM_080457.4 | M-MUC4-S | TGCCTACCAAGTTCACCCCC |
|  | M-MUC4-A | CTTTGTCCAATAGTTCTGTTTCCC |

**Supplementary Table 2**

Table S2. Changes of reducing sugar contents of GP

| Processes | Total sugar content (mg/mL) | Time | Reducing sugar contents (mg/mL) |
| --- | --- | --- | --- |
| Salivary digestion | 14.2713±0.0182 | 0 min | 0.4493±0.0121b |
|  |  | 5 min | 0.4553±0.0147b |
| Gastric digestion |  | 1 h | 0.5062±0.0119a |
|  |  | 2 h | 0.5149±0.0128a |
|  |  | 4 h | 0.5187±0.0014a |
|  |  | 6 h | 0.5199±0.0133a |
| Small intestinal digestion |  | 1 h | 0.5276±0.0147a |
|  |  | 2 h | 0.5311±0.0079a |
|  |  | 4 h | 0.5331±0.0167a |
|  |  | 6 h | 0.5364±0.0126a |
